# Timely expression of PGAM5 and its cleavage control mitochondrial homeostasis during neurite re-growth after traumatic brain injury

**DOI:** 10.1186/s13578-023-01052-0

**Published:** 2023-05-23

**Authors:** Min-Zong Liang, Ting-Hsuan Lu, Linyi Chen

**Affiliations:** 1 Institute of Molecular Medicine, National Tsing Hua University, Hsinchu, Taiwan; 2 Department of Medical Science, National Tsing Hua University, Hsinchu, Taiwan

**Keywords:** Traumatic brain injury, PGAM5, Mitochondrial homeostasis, Neurite re-growth, Epigenetic regulation

## Abstract

**Background:**

Patients suffered from severe traumatic brain injury (TBI) have twice the risk of developing into neurodegenerative diseases later in their life. Thus, early intervention is needed not only to treat TBI but also to reduce neurodegenerative diseases in the future. Physiological functions of neurons highly depend on mitochondria. Thus, when mitochondrial integrity is compromised by injury, neurons would initiate a cascade of events to maintain homeostasis of mitochondria. However, what protein senses mitochondrial dysfunction and how mitochondrial homeostasis is maintained during regeneration remains unclear.

**Results:**

We found that TBI-increased transcription of a mitochondrial protein, phosphoglycerate mutase 5 (PGAM5), during acute phase was via topological remodeling of a novel enhancer-promoter interaction. This up-regulated PGAM5 correlated with mitophagy, whereas presenilins-associated rhomboid-like protein (PARL)-dependent PGAM5 cleavage at a later stage of TBI enhanced mitochondrial transcription factor A (TFAM) expression and mitochondrial mass. To test whether PGAM5 cleavage and TFAM expression were sufficient for functional recovery, mitochondrial oxidative phosphorylation uncoupler carbonyl cyanide 4-(trifluoromethoxy) phenylhydrazone (FCCP) was used to uncouple electron transport chain and reduce mitochondrial function. As a result, FCCP triggered PGAM5 cleavage, TFAM expression and recovery of motor function deficits of CCI mice.

**Conclusions:**

Findings from this study implicate that PGAM5 may act as a mitochondrial sensor for brain injury to activate its own transcription at acute phase, serving to remove damaged mitochondria through mitophagy. Subsequently, PGAM5 is cleaved by PARL, and TFAM expression is increased for mitochondrial biogenesis at a later stage after TBI. Taken together, this study concludes that timely regulation of PGAM5 expression and its own cleavage are required for neurite re-growth and functional recovery.

**Supplementary Information:**

The online version contains supplementary material available at 10.1186/s13578-023-01052-0.

## Background

Traumatic brain injury (TBI) is a critical public health problem with high mortality and morbidity. Approximately 70 million individuals worldwide reported suffer from TBI every year [1]. TBI leads to long-term or permanent disabilities in patients including neurologic dysfunction, neuroendocrine disorders, and psychiatric diseases [2–5]. However, there is currently no effective clinical therapy to promote neurite re-growth after TBI. Mitochondria produces ATP to maintain energy metabolism in neurons. TBI alters mitochondrial homeostasis and results in damage and dysfunction of mitochondria [6–8]. After injury, the overproduction of reactive oxygen species leads to loss of mitochondrial membrane potential (ΔΨ_m_) [9–11]. Besides, TBI increases fragmented mitochondria and expression of dynamin-related protein 1 (DRP1) in injured hippocampal neurons, indicating an up-regulation of mitochondrial fission after TBI [8, 12]. TBI-induced mitochondrial fission mediates mitophagy to eliminate damaged mitochondria and prevent neuronal apoptosis in early-stage of TBI. Inhibition of mitochondrial fission and mitophagy repressed neurite re-growth and aggravated TBI-induced behavioral deficits, suggesting that mitophagy is required to remove damaged mitochondria after TBI [12–14].

While mitophagy prevent neuronal apoptosis, it has been reported that sufficient mitochondria are required to sustain neuronal growth [15]. Induction of mitochondrial biogenesis increased hippocampal functional connectivity and restored neurological function of rats after TBI [16, 17]. Overexpression of peroxisome proliferator-activated receptor gamma coactivator-1 alpha (PGC1α), a major regulator of mitochondrial biogenesis, increased ATP production and axonal length of cortical neurons [15]. Our previous study showed that transplantation of exogenous mitochondria restored ΔΨm and enhanced neurite re-growth of injured hippocampal neurons [12]. Mitochondrial transplantation also enhanced cognitive and motor function recovery of TBI mice [18]. These studies indicate the importance of generating sufficient mitochondria for neurite re-growth. Nonetheless, the regulation of endogenous mitochondrial biogenesis in injured neurons after TBI remains unclear.

Mitochondrial phosphoglycerate mutase family member 5 (PGAM5) has emerged to be a crucial player to maintain mitochondrial homeostasis [18]. The N-terminal of PGAM5 contains a transmembrane domain, which localizes PGAM5 on mitochondrial inner membrane [19, 20]. The C-terminal of PGAM5 contains a phosphatase domain, which recruits DRP1 to mitochondria and dephosphorylates DRP1 at S637 to promote mitochondrial fission [21–23]. When mitochondria are damaged, PGAM5 stabilizes phosphatase and tensin homolog-induced putative kinase protein 1 (PINK1) on mitochondria, leading to PINK1/Parkin-dependent mitophagy [19, 24]. Loss of PGAM5 leads to impaired mitophagy and dopaminergic neurodegeneration [25, 26]. Besides, upon the loss of ΔΨ_m_ of damaged mitochondria, PGAM5 is cleaved at the transmembrane domain by rhomboid protease presenilin-associated rhomboid-like protein (PARL) [19]. Then cleaved PGAM5 is released from mitochondria to cytosol. Cleaved PGAM5 is known to activate WNT signaling through dephosphorylating β-catenin and subsequently enhance target genes for mitochondrial biogenesis in muscle cells and induced pluripotent stem cells [27, 28]. Since PGAM5 regulates mitophagy and mitochondrial biogenesis under stress condition, we hypothesize that PGAM5 governs the interplay between mitochondrial homeostasis and neurite re-growth after TBI.

## Results

### Transcriptional regulation of brain injury-induced PGAM5

**Fig. 1:**
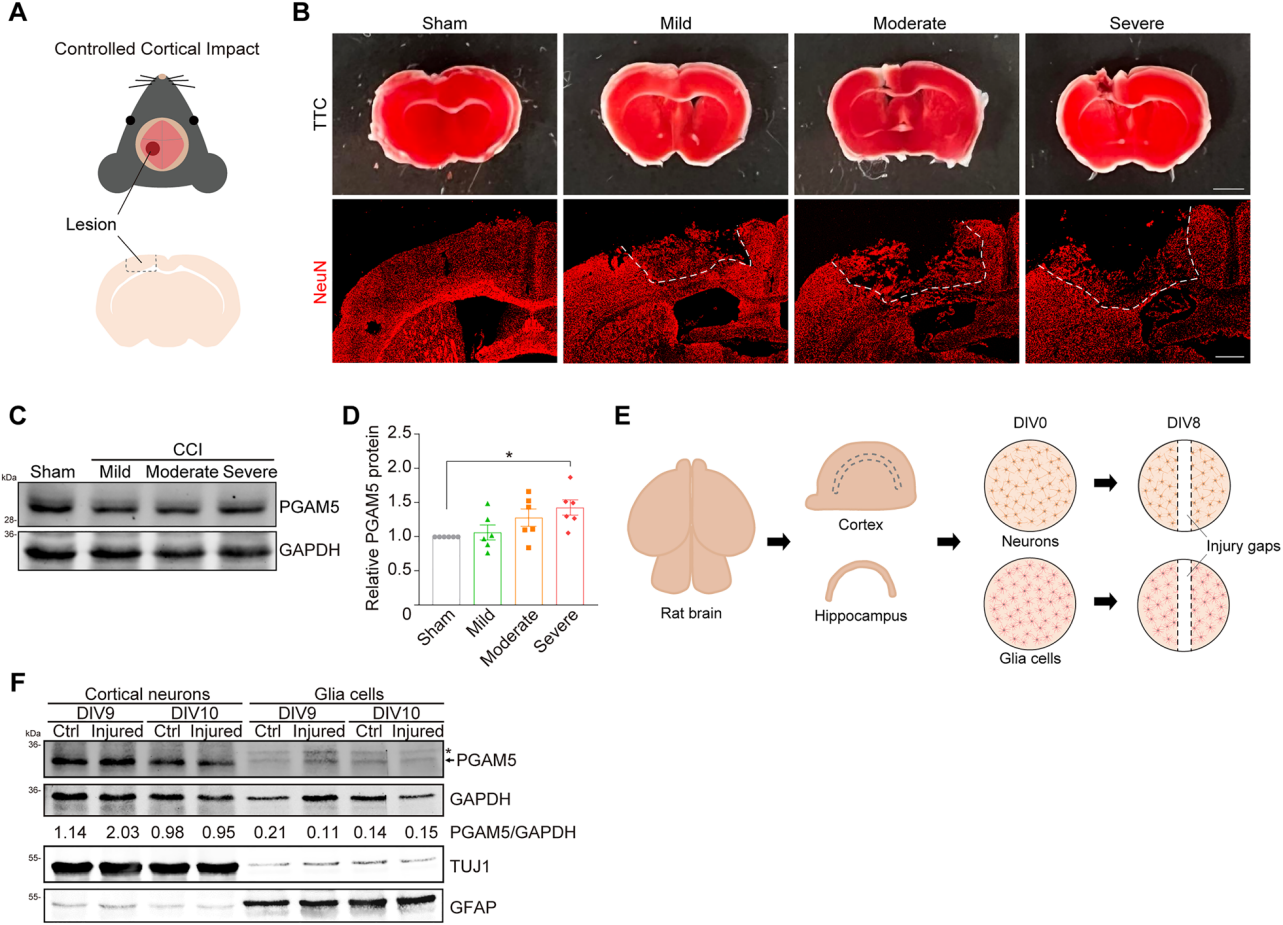
PGAM5 is up-regulated in response to TBI **A** C57BL/6 J male mice were anesthetized and the dura mater of left hemisphere was exposed by a craniectomy. The sensorimotor cortex was impacted by CCI device at different velocity and deformation depth to mimic mild, moderate and severe TBI. **B** TTC staining and immunostaining of NeuN proteins of CCI mouse brains on 4 dpi. White dashed lines indicate the boundaries of the impacted lesion. Scale bar, 2 mm (upper) and 0.5 mm (bottom). **C** Immunoblot of PGAM5 in tissue lysate collected from left hemisphere on 4 dpi. **D** Quantification of relative PGAM5 levels normalized to GAPDH levels. Data are presented as mean ± SEM (n = 6). * *p* < 0.05, one‑way ANOVA with Tukey’s multiple comparisons. **E** Rat cortical neurons, hippocampal neurons and glia cells were isolated and cultured in vitro on DIV0. Primary neurons were scratch-injured with a pipette on DIV 8. Injured area was between the two black dashed lines. **F** Immunoblot of PGAM5 proteins in primary cortical neurons and glia cells. The numbers indicate the relative PGAM5 level normalized to GAPDH. Asterisk indicates the non-specific bands

To investigate whether TBI affects the expression of PGAM5, controlled cortical impact (CCI) on C57BL/6J mice was established as an in vivo TBI model [29, 30]. The left hemisphere of mouse brain was injured by a CCI device at the velocity of 3–5 m/s and the deformation depth of 1–2 mm to mimic mild, moderate and severe TBI (Fig. 1A, B). The injured volume is approximately 2.4% of the half brain. Based on immunoblots of PGAM5, we observed an increase of PGAM5 protein in mice brains after severe CCI injury, compared to the sham group (Fig. 1C, D). Brain contains neurons and glia cells. To determine whether PGAM5 is increased in neurons or glia cells after TBI, we assessed PGAM5 level in primary cortical neurons and glia cells. Rat cortical neurons, hippocampal neurons and glia cells were isolated from embryonic day 18 (E18) rat embryos and cultured on the day in vitro 0 (DIV0) and scratched injured using a pipette tip on DIV8 to mimic TBI-induced injury in vitro (Fig. 1E). PGAM5 protein was increased in injured cortical neurons on DIV9, compared to control neurons (Ctrl). On the other hand, the expression of PGAM5 was relatively low in both un-injured and injured glia cells (Fig. 1F). To determine whether PGAM5 is increased at the transcriptional level, the expression of *Pgam5* gene was examined via semi-quantitative PCR (qPCR) analysis. The relative *Pgam5* mRNA level showed an increased trend in injured cortical neurons compared to non-injury controls (Additional file 1: Fig. S1). Our in vitro injury assays injured only 10% cortical neurons, thus, the observed increase of *Pgam5* is likely under-estimated. These results suggest that the increased PGAM5 expression correlates with severity of brain injury and PGAM5 is expressed predominantly in neurons.

**Fig. 2:**
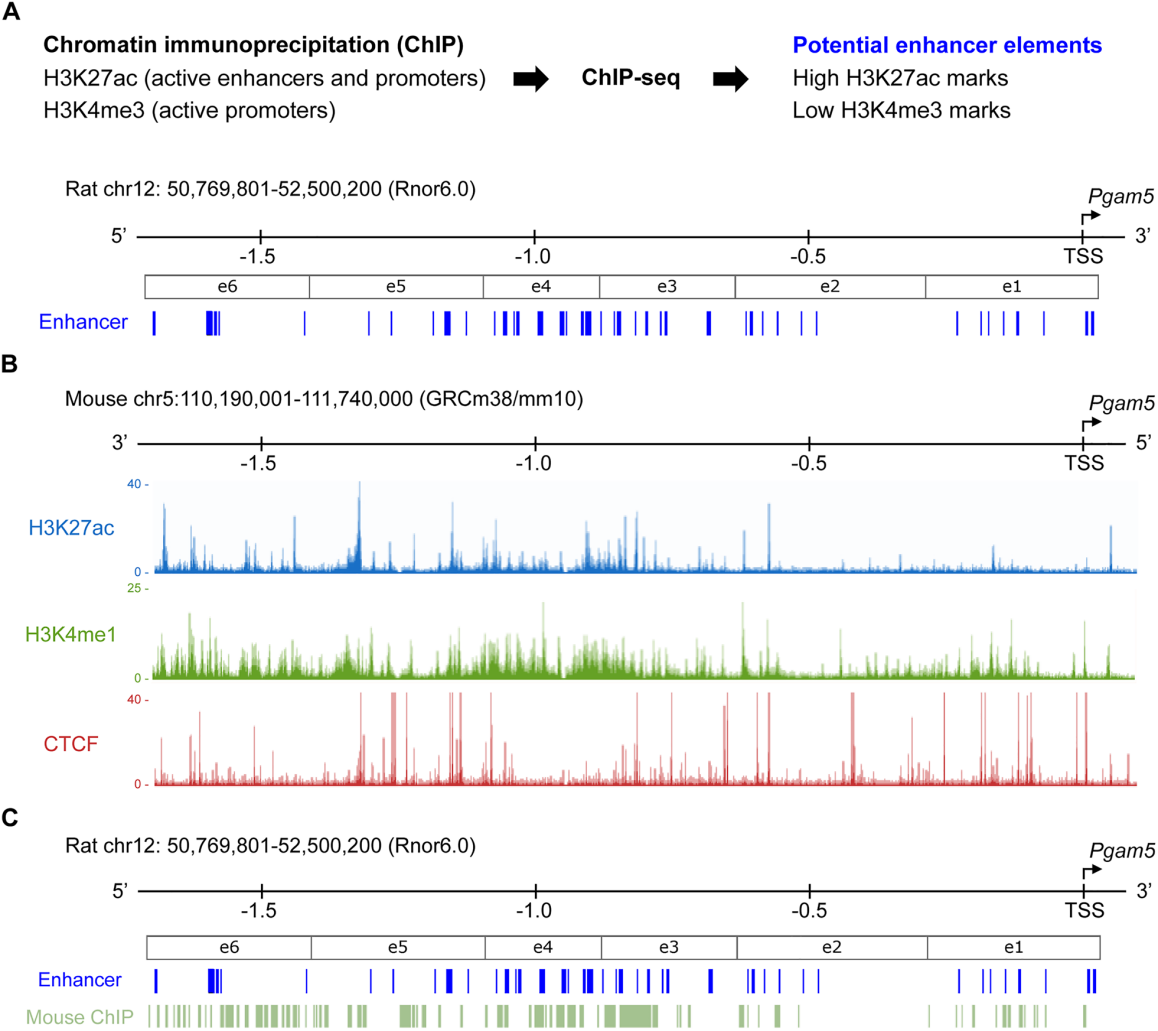
Putative enhancer regions of *Pgam5* in rat genome **A** Putative enhancer elements of *Pgam5* (blue bars) in the rat genome (Rnor6.0) were predicted using our previously developed algorism based on ChIP-seq results of H3K27ac and H3K4me3. Putative enhancer elements were divided into 6 genomic regions, from e1 to e6. TSS, transcription start site. **B** ChIP-seq data of H3K27ac, H3K4me1 and CTCF in homologous loci of putative enhancer elements in mouse genome from the ENCODE database. **C** Putative enhancer elements (blue) aligned with potential active enhancer regions in homologous loci predicted by mouse ChIP-seq results (green)

We next investigated how PGAM5 level was regulated in response to injury. Since the enhancer for *Pgam5* transcription has not been reported, we predicted putative enhancer element of *Pgam5* using our previously developed algorism based on specific histone marks, histone H3 lysine 27 acetylation (H3K27ac) and histone H3 lysine 4 tri-methylation (H3K4me3). As shown in Fig. 2A, the 1.7 Mb flanking the transcription start site of *Pgam5* in the rat genome (Rnor6.0) was predicted as a putative enhancer and further divided into six genomic sub-regions, e1-e6 [31]. To verify which sub-region is the dominant enhancer region of *Pgam5*, we compared the chromatin immunoprecipitation-sequencing data of enhancer marks, H3K27ac, histone H3 lysine 4 monomethylation (H3K4me1) and CTCF binding sites of mouse genome from the ENCODE database (Fig. 2B) [32–34]. The regions enriched with H3K27ac, H3K4me1 and CTCF signals were aligned back to the rat genome to map the putative enhancer regions (Fig. 2C).

**Fig. 3:**
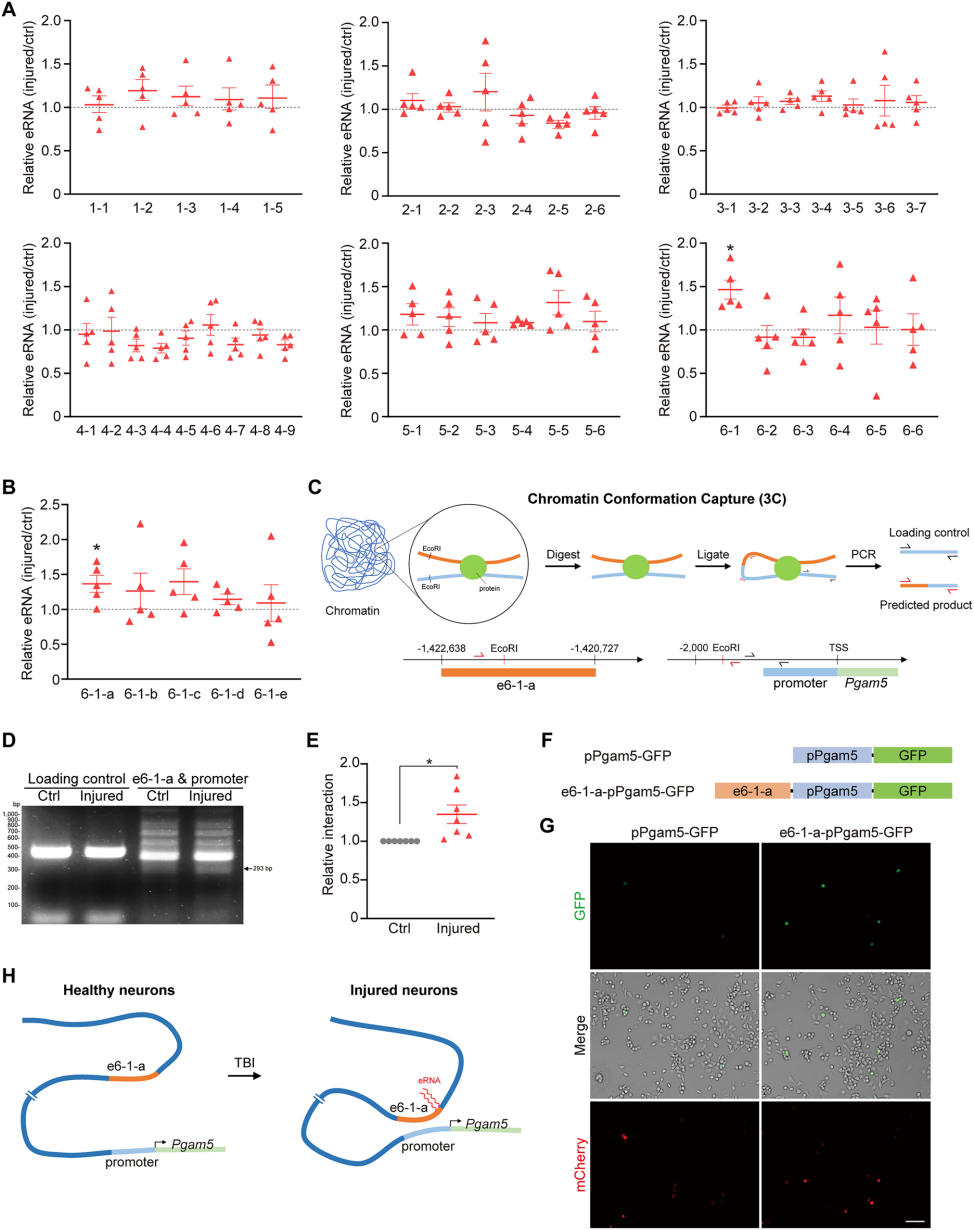
Active enhancer sub-region e6-1-a interacts with *Pgam5* promoter after TBI **A** Relative enhancer RNA expression of putative enhancers sub-regions in injured cortical neurons on DIV9, normalized to un-injured groups. **B** Relative enhancer RNA expression of e6-1 sub-regions in injured cortical neurons on DIV9. The enhancer RNA expression is assessed by PCR. Grey dashed lines indicate fold change equal to 1. Data are presented as mean ± SEM (n = 5). * *p* < 0.05, Student’s t-test. **C** Schematic flow chart of 3C assays. The interaction between e6-1-a region and *Pgam5* promoter was assessed by 3C assays. Red arrows indicate primers for predicted ligation product. Black arrows indicate primers for loading control. EcoRI: Restriction site of EcoRI. TSS, transcription start site. **D** Electrophoresis of predicted PCR products of 3C assays. The length of PCR products of loading control and predicted ligation product are 457 bp and 293 bp, respectively. Arrow indicates the band of predicted ligation product of 3C assays. **E** Quantification of relative intensity of predicted ligation product in (**D**), normalized to loading control. Data are presented as mean ± SEM (n = 7). * *p* < 0.05, Student’s t-test. **F** Constructs of pPgam5-GFP and e6-1-a-pPGAM5-GFP. pPgam5: *Pgam5* promoter. **G** Neuro2a cells were transiently transfected with pPgam5-GFP or e6-1-a-pPgam5-GFP constructs, together with pmCherry-expressing plasmid, for 24 h. Fluorescence images were taken using Carl Zeiss Observer Z1 microscope. Scale bar, 100 μm. **H** Schematic model of genomic architecture transformation of enhancer-promoter looping of *Pgam5* after TBI

The transcript of enhancer RNA (eRNA) is regarded as one of the hallmarks of active enhancers because it interacts with transcriptional regulators and stabilizes enhancer-promoter looping to regulate gene expression [35–37]. To determine whether putative enhancer sub-regions of *Pgam5* were active in response to TBI, the expression of eRNAs derived from putative enhancer regions were quantified. We observed a significant increase of eRNA level at enhancer sub-region e6-1 in injured cortical neurons on DIV9, compared to un-injured neurons (Fig. 3A). We further divided e6-1 to e6-1-a to e6-1-e. The relative eRNA level of e6-1-a in injured cortical neurons on DIV9 was increased compared to no injury control, suggesting that e6-1-a is likely an active enhancer during TBI-induced *Pgam5* transcription (Fig. 3B). To investigate how a distant enhancer can regulate transcription, we examined the possible looping interaction between e6-1-a and promoter of *Pgam5*. To this end, we performed chromosome conformation capture (3C) assays to assess genomic topology as describe in Fig. 3C. The interaction between e6-1-a enhancer region and *Pgam5* promoter was significantly up-regulated in injured cortical neurons, compared to un-injured neurons (Fig. 3D, E). To further determine whether the interaction between e6-1-a and *Pgam5* promoter regulates *Pgam5* expression, we engineered a *Pgam5* promoter (pPgam5)-driven GFP expression construct (pPgam5-GFP) and an enhancer-promoter-driven GFP reporter (e6-1-a-pPgam5-GFP) construct (Fig. 3F). Neuro2a cells were transiently transfected with either of these two constructs, along with a pmCherry-expressing plasmid to indicate similar transfection efficiency in these two conditions. Increased GFP expression was observed in neuro2a cells transfected with e6-1-a-pPgam5-GFP, compared to cells transfected with pPgam5-GFP, suggesting an increased transcriptional activity by e6-1-a-pPgam5-GFP (Fig. 3G). The bright view images showed similar cell numbers under the imaged fields. This result demonstrated that active enhancer region e6-1-a indeed increased transcriptional activity with pPgam5. Together, these findings reveal that TBI induces the eRNA transcription of e6-1-a and the interaction between e6-1-a and *Pgam5* promoter drives *Pgam5* transcription (Fig. 3H).

### Cleaved PGAM5 enhances neurite re-growth of injured cortical neurons

**Fig. 4:**
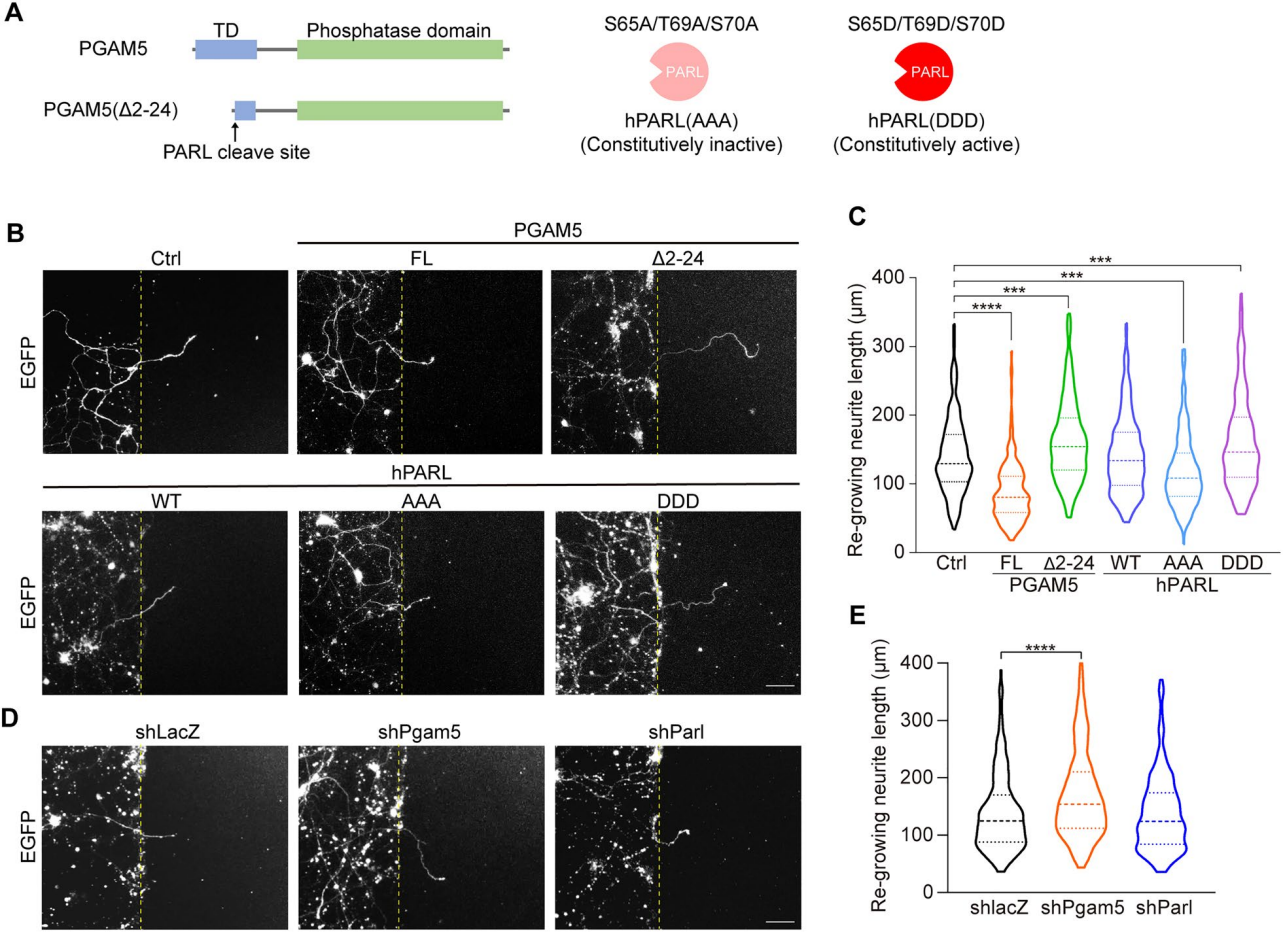
Cleaved PGAM5 enhances neurite re-growth in injured cortical neurons **A** Schematic model of PGAM5, PGAM5(Δ2-24), hPARL(AAA) and hPARL(DDD) constructs. TD, transmembrane domain. **B** Images of injured cortical neurons overexpressing PGAM5, PGAM5(Δ2-24), wild-type hPARL, hPARL(AAA) and hPARL(DDD) on DIV9. Cortical neurons were visualized by co-transfection of EGFP vector. Yellow dashed lines indicate the borders of injured gap. Scale bar, 100 μm. **C** Quantification of the length of re-growing neurites in (**B**). Dashed lines indicate the medium and dotted lines indicate the 25th and the 75th percentiles (n = 209–238 cells/group). *** *p* < 0.001, **** *p* < 0.0001, one‑way ANOVA with Dunnett’s multiple comparisons. **D** Images of injured cortical neurons transiently transfected with shlacZ, shPgam5 or shParl vectors on DIV9. Cortical neurons were visualized by co-transfection of EGFP vector. Yellow dashed lines indicate the borders of injured gap. Scale bar, 100 μm. **E** Quantification of the length of re-growing neurites in (**D**). Dashed lines indicate the medium and dotted lines indicate the 25th and the 75th percentiles (n = 220–273 cells/group). **** *p* < 0.0001, one‑way ANOVA with Dunnett’s multiple comparisons

Next, we investigated whether TBI-induced up-regulation of PGAM5 affects neurite re-growth of injured cortical neurons. PGAM5 can be cleaved by PARL in response to mitochondrial damage, thus it is yet to be determined whether full length PGAM5 at the mitochondrial inner membrane regulates neurite re-growth or cleaved PGAM5 controls the neurite re-growth. Full-length PGAM5 and truncated PGAM5(Δ2-24), representing the cleaved form of PGAM5 (Fig. 4A), were transiently overexpressed in cortical neurons and the length of re-growing neurites were measured 24 h after injury. Overexpression of full-length PGAM5 reduced the length of re-growing neurites but PGAM5(Δ2-24) significantly increased the length of re-growing neurites, compared to EGFP control group (Fig. 4B, C). This data demonstrates that full-length PGAM5 represses and cleaved PGAM5 enhances neurite re-growth, respectively. We then hypothesized that induction of PGAM5 cleavage by overexpression of protease PARL promotes neurite re-growth after TBI. To verify this hypothesis, wild-type human PARL (hPARL), constitutively inactive hPARL(S65A/T69A/S70A), hPARL(AAA), and constitutively active hPARL(S65D/T69D/S70D), hPARL(DDD) [38–40], were transiently overexpressed in cortical neurons (Fig. 4A). Overexpression of hPARL did not affect the length of re-growing neurites, compared to EGFP control group. In contrast, overexpression of hPARL(DDD), mimicking phosphorylated PARL with higher activity [37–39], promoted neurite re-growth of injured cortical neurons. Alternatively, overexpression of hPARL(AAA) reduced the length of re-growing neurites (Fig. 4B, C). These results indicate that activation of PARL and cleaved PGAM5 enhance neurite re-growth but full-length PGAM5 represses neurite re-growth. Likewise, the length of re-growing neurites was increased in PGAM5 knockdown cortical neurons (Fig. 4D, E). Neither overexpression nor knockdown of PARL affects neurite re-growth, suggesting that the increase in activity of PARL, but not expression of PARL, is beneficial to neurite re-growth after TBI (Fig. 4B, C, D, E).

### Full-length PGAM5 induces mitophagy

**Fig. 5:**
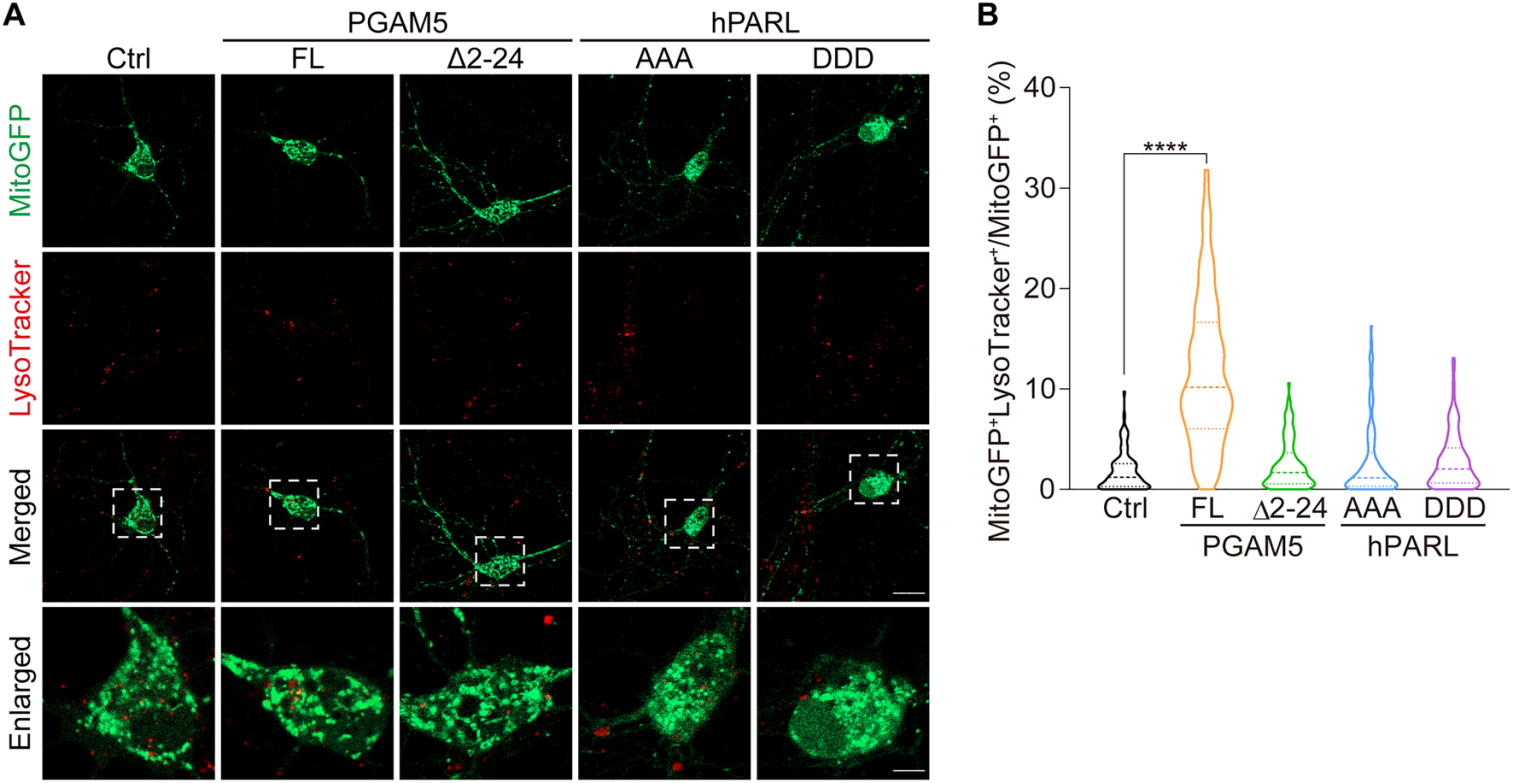
Overexpressing full-length PGAM5 correlates with mitophagy in hippocampal neurons **A** Images of hippocampal neurons overexpressing PGAM5, PGAM5(Δ2-24), hPARL(AAA) or hPARL(DDD). Mitochondria and lysosomes were labeled by transient expression of MitoGFP and LysoTracker staining, respectively. Enlarged, enlarged images of the regions indicated in dashed square in the merged images. Scale bar, 20 μm (merged images) and 5 μm (enlarged images). **B** Quantification of co-localization% of mitochondria and lysosomes in individual hippocampal neurons. FL, full-length. WT, wild type. Dashed lines indicate the medium and dotted lines indicate the 25th and the 75th percentiles (n = 105 cells/group). **** *p* < 0.0001, one‑way ANOVA with Dunnett’s multiple comparisons

TBI increased the expression of full-length PGAM5 (Fig. 1C, D). However, increased full-length PGAM5 repressed neurite re-growth in injured cortical neurons (Fig. 4B, C). The fact that PGAM5 has been shown to mediate PINK1-mitophagy process [26, 28], we hypothesized that full-length PGAM5 may induce mitophagy, leading to poor neurite re-growth. To determine whether PGAM5 would induce mitophagy in neurons, mitochondria and lysosomes were labeled by transiently expressing MitoGFP and LysoTracker staining, respectively. Mitophagy in individual hippocampal neurons was quantified by the co-localization percentage of mitochondria and lysosomes. We observed an increased co-localization of mitochondria and lysosomes in hippocampal neurons overexpressing full-length PGAM5, compared to control group (Fig. 5A, B). Co-localization percentage of mitochondria and lysosomes in hippocampal neurons were not changed after overexpression of PGAM5(Δ2-24), hPARL(AAA) or hPARL(DDD), suggesting that the increase in full-length PGAM5, but not cleaved PGAM5, enhanced mitophagy in neurons (Fig. 5A, B). Thus, overexpression of PGAM5 possibly causes excessive mitophagy in neurons and leads to poor neurites re-growth. In the case of injured neurons, mitophagy may serve to remove damaged mitochondria [13, 14], nonetheless, sufficient amount of mitochondria are required for neurite re-growth [12, 15].

### Cleaved PGAM5 enhances TFAM expression and mitochondrial biogenesis

**Fig. 6:**
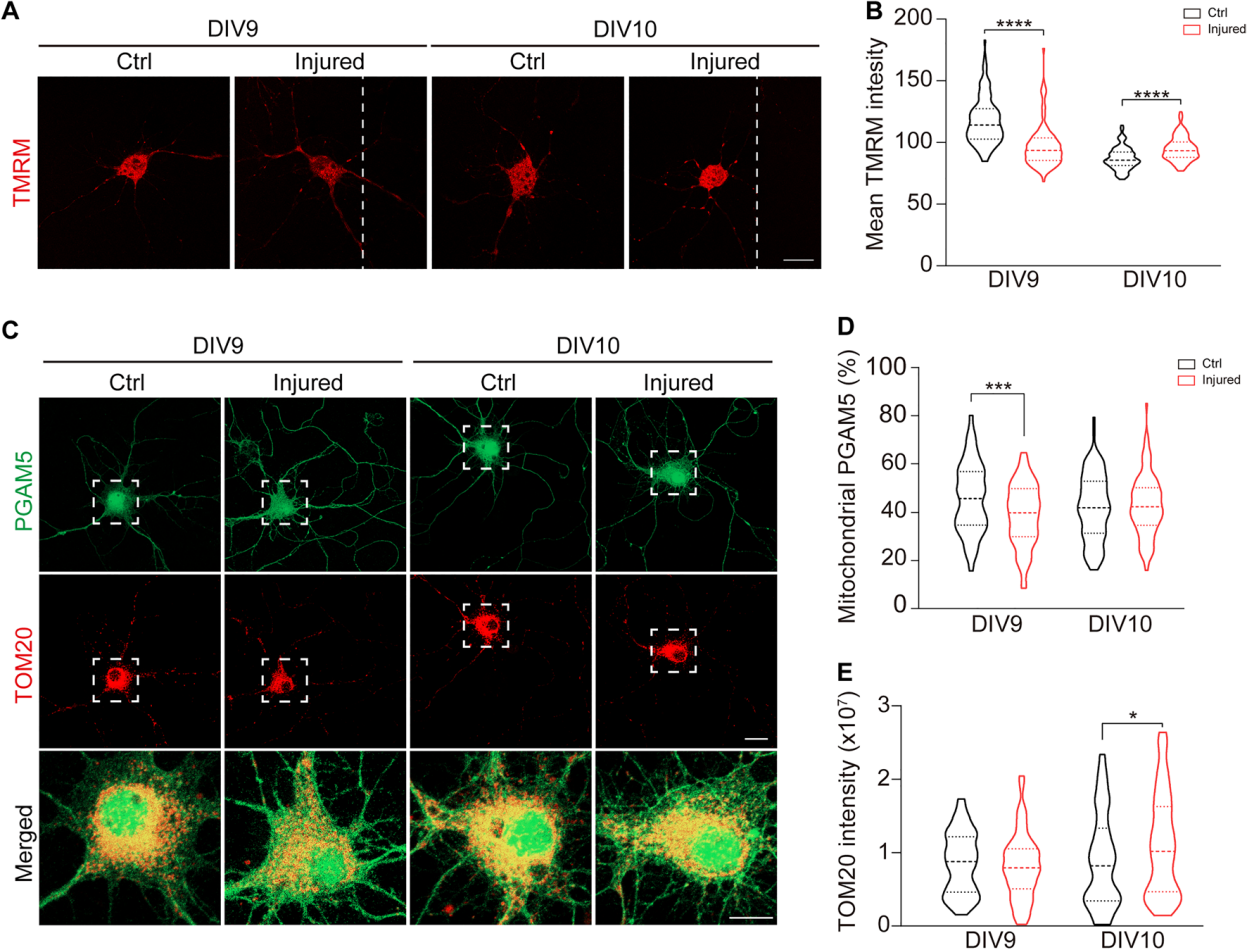
TBI induces loss of ΔΨ_m_ and reduces mitochondrial PGAM5 **A** Mitochondrial membrane potential of hippocampal neurons was accessed by TMRM staining. White dashed lines indicate the borders of the injured gap. Scale bar, 50 µm. **B** Quantification of mean intensity of TMRM in individual hippocampal neurons. Dashed lines indicate the medium and dotted lines indicate the 25th and the 75th percentiles (n = 115–127 neurons/group). **** *p* < 0.0001, unpaired t-test. **C** Immunofluorescence of PGAM5 (green) and mitochondrial outer membrane protein TOM20 (red) in hippocampal neurons. Scale bar, 20 µm (TOM20 staining) and 10 µm (merged images). **D** The percentage of mitochondrial PGAM5 was quantified by PGAM5^+^TOM20^+^ area/PGAM5^+^ area. **E** Quantification of TOM20 intensity in hippocampal neurons. Dashed lines indicate the medium and dotted lines indicate the 25th and the 75th percentiles (n = 110 neurons/group). * *p* < 0.05, *** *p* < 0.001, unpaired t-test

Our results showed that cleaved PGAM5 enhanced neurite re-growth in injured cortical neurons (Fig. 4B, C). PGAM5 is cleaved and released from mitochondria to cytosol in response to loss of ΔΨ_m_ [19]. Since TBI induces mitochondrial damage [10, 11], we examined whether TBI reduces ΔΨ_m_ in injured neurons, leading to the release of cleaved PGAM5. To determine whether TBI affected ΔΨ_m_, tetramethylrhodamine methyl ester (TMRM) was used to label active mitochondria in cortical neurons because it accumulates in active mitochondria with intact ΔΨ_m_. TMRM intensity was significantly reduced in injured neurons compared to un-injured neurons on DIV9, indicating that ΔΨ_m_ was reduced during acute phase of TBI. Subsequently, ΔΨ_m_ in injured neurons was recovered on DIV10 (Fig. 6A, B). This data demonstrates that ΔΨ_m_ is reduced in injured neurons at early-stage after TBI, followed by an increase of ΔΨ_m_ back to steady-state.

**Fig. 7:**
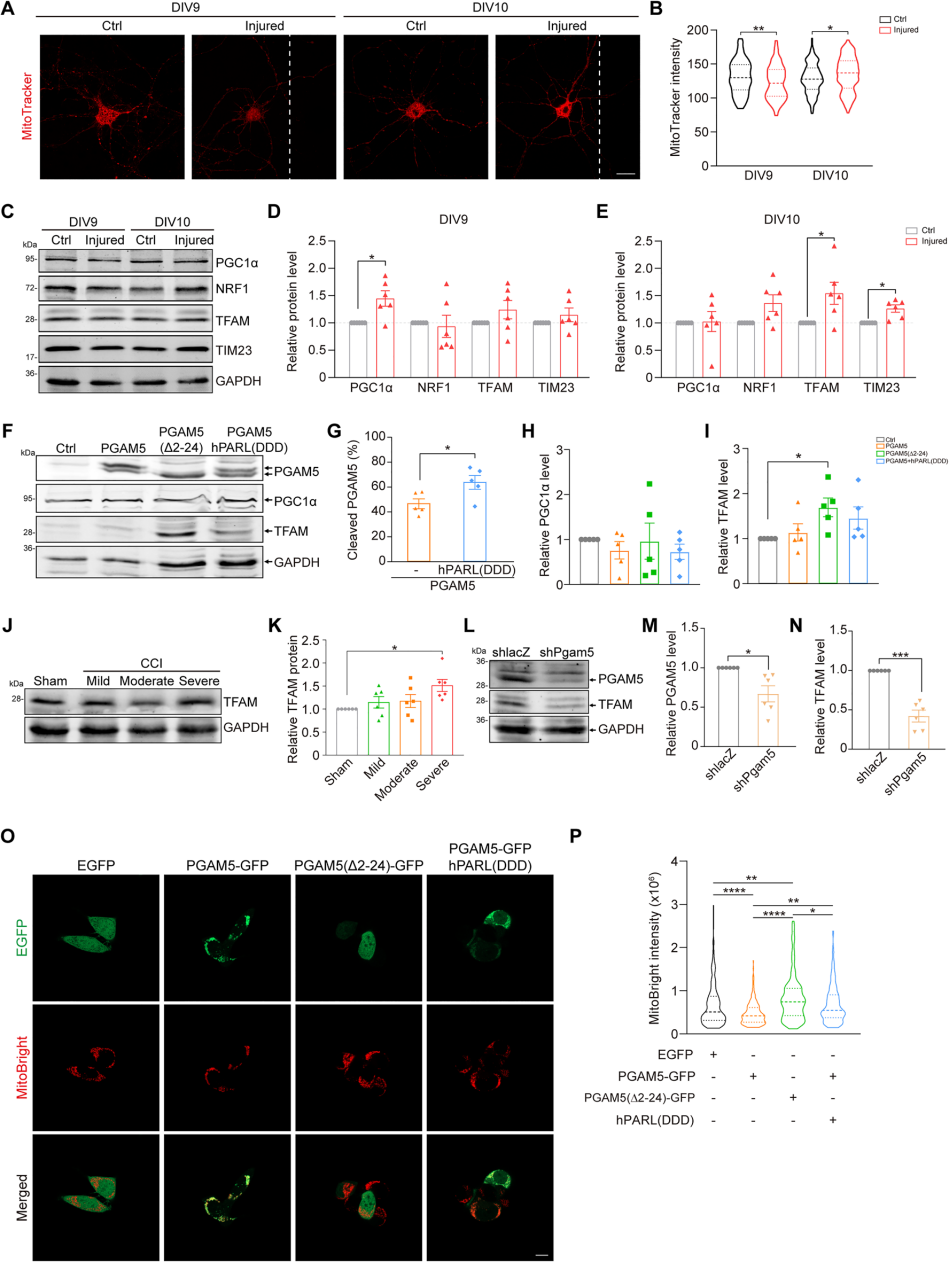
Cleaved PGAM5 enhances mitochondrial biogenesis **A** Mitochondria were visualized by MitoTracker Red staining in hippocampal neurons. Scale bar, 20 µm. **B** Quantification of total MitoTracker intensity in individual neurons. Dashed lines indicate the medium and dotted lines indicate the 25th and the 75th percentiles (n = 181–190 cells/group). * *p* < 0.05, ** *p* < 0.01, Student’s t-test. **C** Immunoblot of PGC1α, NRF1, TFAM and TIM23 in control and injured cortical neurons. **D**, **E** Quantification of (**C**) on DIV9 and DIV10 respectively. Data are presented as mean ± SEM (n = 6). * *p* < 0.05, Student’s t-test. **F** Immunoblot of PGC1α, PGAM5 and TIM23 in neuro2a cells overexpressing full-length PGAM5, PGAM5(Δ2-24) or full-length PGAM5 + hPARL(DDD). **G** Percentage of cleaved PGAM5 in neuro2a cells overexpressing full-length PGAM5 with or without co-expressing hPARL(DDD). Data are presented as mean ± SEM (n = 5). * *p* < 0.05, paired t-test. **H**, **I** Quantification of (**F**). Data are presented as mean ± SEM (n = 5). * *p* < 0.05, one‑way ANOVA with Dunnett’s multiple comparisons. **J** Immunoblot of TFAM in brain tissue lysate of CCI mice on 4 dpi. **K** Quantification of relative TFAM in **J**. Data are presented as mean ± SEM (n = 6). * *p* < 0.05, one‑way ANOVA with Tukey’s multiple comparisons. **L** Immunoblot of PGAM5 and TFAM in neuro2a cells transiently transfected with shlacZ or shPgam5 vector. **M**, **N** Quantification of relative PGAM5 and TFAM in (**L**). Data are presented as mean ± SEM (n = 6). * *p* < 0.05, *** *p* < 0.001, paired t-test. **O** Images of neuro2a cells overexpressing EGFP, PGAM5-GFP, PGAM5(Δ2-24)-GFP or PGAM5-GFP + hPARL(DDD). Mitochondria were visualized by MitoBright LT Deep Red. Scale bar, 20 μm. **P** Quantification of total intensity of MitoBright LT Deep Red in individual cells. Dashed lines indicate the medium and dotted lines indicate the 25th and the 75th percentiles (n = 188–194 cells/group). * *p* < 0.05, ** *p* < 0.01, **** *p* < 0.0001, one‑way ANOVA with Tukey’s multiple comparisons

As ΔΨ_m_ was decreased 24 h after TBI, it is possible that cleaved PGAM5 would be released from mitochondria after injury. Indeed, we found a decrease in the percentage of mitochondrial PGAM5 on DIV9 (Fig. 6C, D) based on the immunofluorescence staining of PGAM5 and mitochondrial outer membrane protein TOM20, corresponding to TBI-induced loss of ΔΨ_m_ (Fig. 6A, B). At a later stage of TBI, an increase in TOM20 intensity was found in injured hippocampal neurons 48 h after TBI (Fig. 6E), implicating an increase of mitochondria. It was reported that increased mitochondrial density was critical for re-growth of injured neurons in *Caenorhabditis elegans* [41]*.* We hypothesized that the increased mitochondria at later stage of TBI was correlated with neurite re-growth. To further determine whether mitochondria were increased in injured hippocampal neurons, mitochondria were visualized through MitoTracker staining. We observed a decrease in mitochondria in injured hippocampal neurons 24 h after injury, compared to un-injured neurons (Fig. 7A, B). Since PGAM5 was up-regulated after TBI (Fig. 1C, D) and increased PGAM5 enhanced mitophagy (Fig. 5A, B), we reasoned that the decreased mitochondria in injured neurons was possibly caused by PGAM5-induced mitophagy. In contrast, MitoTracker intensity was restored in injured hippocampal neurons on DIV10 (Fig. 7A, B), suggesting that the amount of mitochondria was restored 48 after TBI. To determine whether mitochondrial biogenesis is responsible for this increased amount of mitochondria, we assessed the expression of PGC1α, nuclear respiratory factor 1 (NRF1) and mitochondrial transcription factor A (TFAM), which are master regulators of mitochondrial biogenesis, in injured cortical neurons. We found that PGC1α protein was significantly increased in injured cortical neurons on DIV9, followed by an increase of TFAM protein DIV10. Along the same line, mitochondrial inner membrane protein TIM23 was increased in injured cortical neurons on DIV10 (Fig. 7C, D, E). Based on these findings, increased mitochondrial mass is likely through subsequent increase of PGC1α and TFAM after TBI.

Our results revealed that the percentage of mitochondrial PGAM5 was decreased in injured neurons (Fig. 6D), suggesting the possibility that TBI causes the release of cleaved PGAM5 from mitochondria. Given the time sequence of cleaved PGAM5 and increased mitochondria mass, we hypothesized that cleaved PGAM5 leads to mitochondrial biogenesis. To this end, PGAM5(Δ2-24) was transiently overexpressed in mouse neuroblastoma neuro2a cells. Based on immunoblots of PGC1α and TFAM, we found that overexpression of PGAM5(Δ2-24), rather than full-length PGAM5, increased TFAM protein in neuro2a cells (Fig. 7F, H, I). In addition, TFAM protein was increased in mice brain after severe CCI injury, suggesting that cleaved PGAM5 may correlate with TFAM expression (Fig. 7J, K). Moreover, TFAM protein was reduced in PGAM5-knockdown neuro2a cells compared with shlacZ control cells (Fig. 7L, M, N), suggesting a PGAM5-dependent regulation of TFAM level. These results demonstrate that cleaved PGAM5 increases expression of TFAM, which has been implicated in replication of mitochondrial DNA [42].

To directly correlates cleaved PGAM5 and increases mitochondria in neurons, mitochondria were visualized via MitoBright Deep Red staining in neuro2a cells. MitoBright intensity in individual cells was significantly increased in neuro2a cells overexpressing PGAM5(Δ2-24)-GFP, compared to control cells expressing EGFP (Fig. 7O, P). We observed that MitoBright intensity was reduced in cells overexpressing PGAM5-GFP (Fig. 7O, P), likely due to mitophagy (Fig. 5A, B). Full-length PGAM5 localizes on mitochondria inner membrane but cleaved PGAM5 is released from mitochondria to cytosol [19, 27]. Indeed, PGAM5-GFP was co-localized with mitochondria and PGAM5(Δ2-24)-GFP was diffused in cytosol (Fig. 7O). Consistent with this finding, a proportion of PGAM5-GFP translocated to cytosol after co-overexpressing hPARL(DDD) in neuro2a cells, which promoted the cleavage of PGAM5 (Fig. 7F, G, O). In addition, co-overexpression of PGAM5-GFP and hPARL(DDD) increased mitochondria in neuron2a cell, compared to cells expressing PGAM5-GFP only (Fig. 7O, P). These results demonstrate that cleaved PGAM5, rather than full-length PGAM5, enhances mitochondrial biogenesis in neurons. In contrast to a study showing that cleaved PGAM5 induced expression of PGC1α to enhance mitochondrial biogenesis in pluripotent stem cells [28], our results demonstrated that cleaved PGAM5 increased expression of TFAM and mitochondrial biogenesis in injured neurons.

### FCCP induces the cleavage of PGAM5 and promotes motor function recovery

**Fig. 8:**
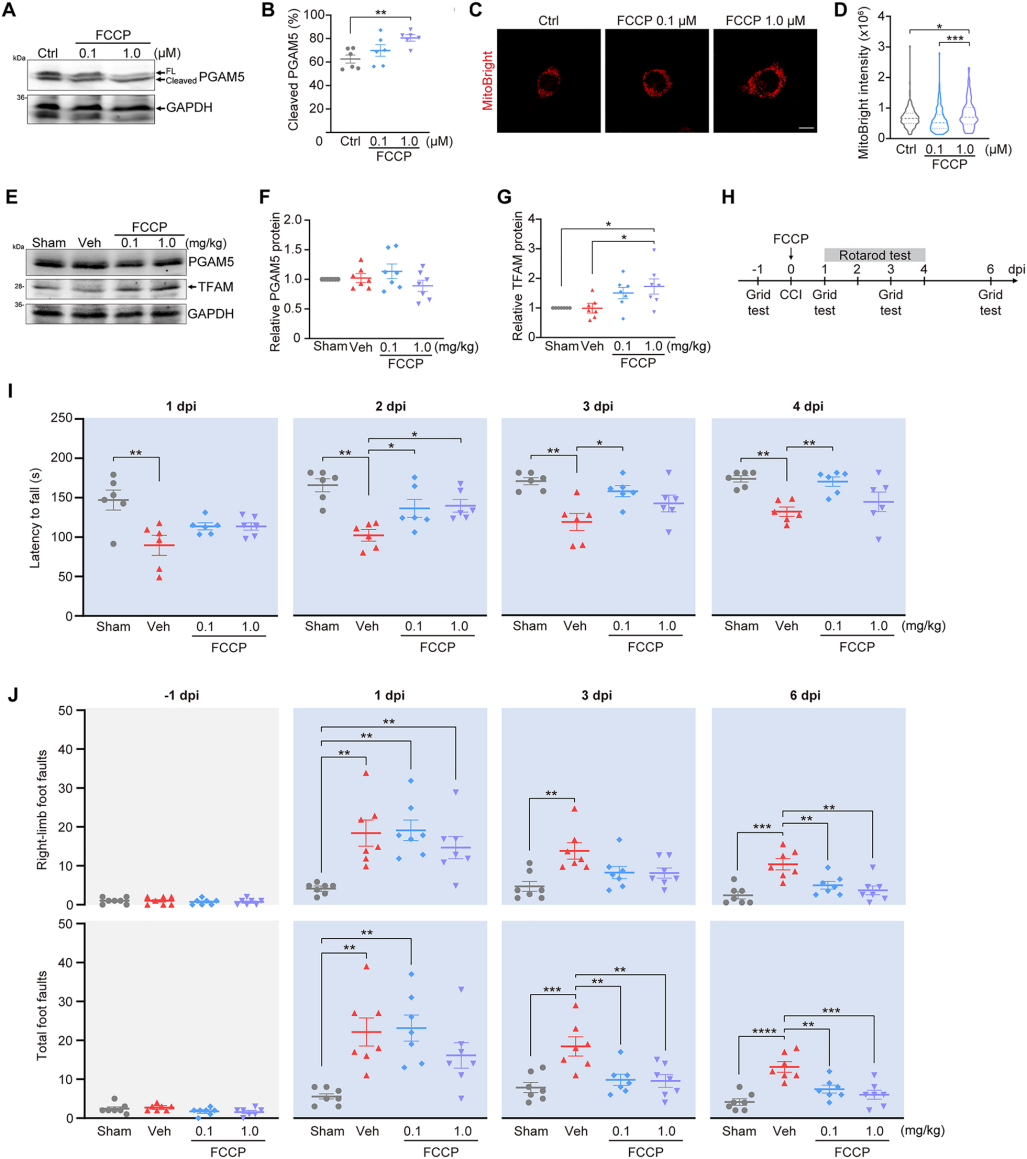
FCCP induces PGAM5 cleavage and promotes motor function recovery of CCI mice **A** Immunoblots of PGAM5 in neuro2a cells treated with DMSO (ctrl), 0.1 μM or 1.0 μM FCCP for 24 h. FL: full-length. **B** The percentage of cleaved PGAM5 was quantified by cleaved PGAM5/total PGAM5. Data are presented as mean ± SEM (n = 6). ** *p* < 0.01, one‑way ANOVA with Tukey’s multiple comparisons. **C** Mitochondria were visualized by MitoBright LT Deep Red in neuro2a cells. Scale bar, 10 μm. **D** Quantification of total intensity of MitoBright in individual neuro2a cells. Dashed lines indicate the medium and dotted lines indicate the 25th and the 75th percentiles (n = 140–159 cells/group). * *p* < 0.05, *** *p* < 0.001, one‑way ANOVA with Dunnett’s multiple comparisons. **E** Immunoblot of PGAM5 and TFAM in mice brain on dpi 7. Veh, vehicle control. **F**, **G** Quantification of PGAM5 and TFAM in (**E**). Data are presented as mean ± SEM (n = 7). * *p* < 0.05, one‑way ANOVA with Tukey’s multiple comparisons. (H) Timeline of the experimental design for rotarod test and grid test. C57BL/6 J mice were intranasally administrated 0.1 mg/kg FCCP, 1.0 mg/kg FCCP or vehicle (DMSO) at 6 h after CCI on 0 dpi. Rotarod test was performed on 1–4 dpi. Grid test was performed on -1, 1, 3 and 6 dpi. **I** Quantification of latency to fall of mice on 1–4 dpi. Data are presented as mean ± SEM (n = 6). * *p* < 0.05, ** *p* < 0.01, one‑way ANOVA with Tukey’s multiple comparisons. **J** Quantification of foot faults of CCI mice on -1, 1, 3 and 6 dpi. Data are presented as mean ± SEM (n = 7). ** *p* < 0.01, *** *p* < 0.001, **** *p* < 0.0001, one‑way ANOVA with Tukey’s multiple comparisons

If PGAM5 cleavage increased mitochondrial mass and promoted neurite re-growth, identifying an approach to increase PGAM5 cleavage would be beneficial to TBI patients. Judged by the fact that PGAM5 cleavage is triggered by mitochondrial damage, mitochondrial oxidative phosphorylation uncoupler carbonyl cyanide 4-(trifluoromethoxy) phenylhydrazone (FCCP) could be a good choice of inducer. depolarizes ΔΨ_m_ and induces mitophagy [43]. To avoid mitophagy, we chose to use low dosage of FCCP to mildly reduce ΔΨ_m_ [44]. As shown in Fig. 8A, B, neuro2a cells were treated with 0.1 μM FCCP, 1.0 μM FCCP or DMSO (control) for 24 h, and the percentage of cleaved PGAM5 was increased in cells treated with 1.0 μM FCCP. We next examined whether FCCP would promote mitochondrial biogenesis. By staining of mitochondria with MitoBright, we found significantly increased mitochondria in neuro2a cells treated with 1.0 μM FCCP (Fig. 8C, D). These results indicate that low-dose FCCP enhances PGAM5 cleavage and mitochondrial biogenesis.

To examine whether FCCP is beneficial for functional recovery of TBI, CCI mice were intranasally administrated 0.1 or 1 mg/kg FCCP at 6 h after CCI injury. Tissue lysate was collected from the left hemisphere of mouse brain (injured hemisphere) on 7 days post injury (dpi). TFAM protein was shown increased in CCI mice after administration of 1.0 mg/kg FCCP, compared to vehicle (DMSO) control mice (Fig. 8E, F, G). This result implicates that FCCP likely increases mitochondrial mass through increasing TFAM. Since the sensorimotor cortex in left hemisphere of mice were injured by CCI, we assessed the motor function of CCI mice through rotarod test and grid test (Fig. 8H). In rotarod test, CCI mice were placed on a rotating rod accelerating from 4 to 40 rpm over 180 s. The time latency to fall from the rotarod was recorded to evaluate motor function of mice [45]. We observed that CCI mice treated with vehicle fell more quickly than sham mice on 1 dpi, indicating that CCI impaired motor function (Fig. 8I). The latency to fall for mice treated with 0.1 or 1.0 mg/kg FCCP were significantly increased on 2 dpi, compared to vehicle group. Additionally, the latency to fall for mice treated with 0.1 mg/kg FCCP was increased on 3 and 4 dpi (Fig. 8I). In grid test, mice were placed on a wire grid for 5 min. The numbers of foot faults were counted to evaluated motor function deficits as well as coordination post injury [46]. The right-limb foot faults of CCI mice treated with vehicle or FCCP were significantly increased on 1 dpi, compared to sham mice (Fig. 8J). This result reflects that CCI induces motor function deficits of contralateral limbs since the sensorimotor cortex in left hemisphere of mice were injured by CCI (Fig. 1A). The right-limb foot faults were decreased on 6 dpi for mice treated with 0.1 or 1.0 mg/kg FCCP compared to vehicle group, suggesting a reduced motor function impairment by FCCP. Similarly, the total foot faults were significantly decreased for mice treated with 0.1 or 1.0 mg/kg FCCP on 3 and 6 dpi (Fig. 8J). These results indicate that low dosages of FCCP enhance motor function recovery of CCI mice.

**Fig. 9:**
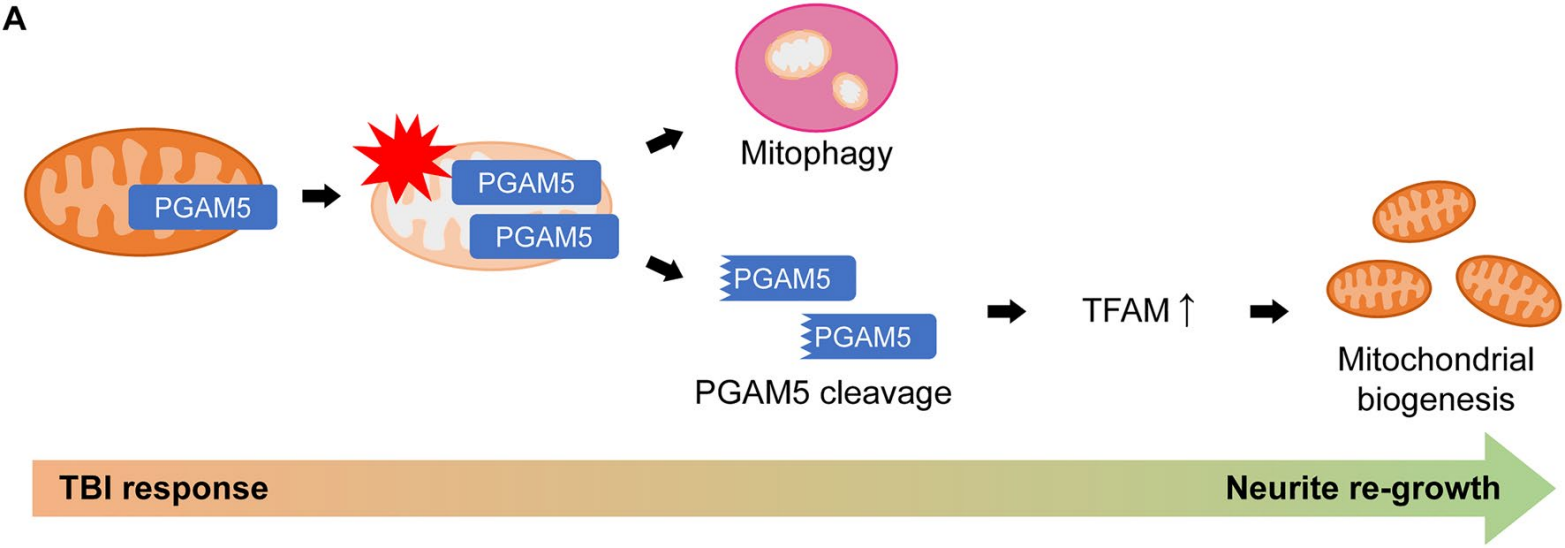
Elevated PGAM5 enhances mitophagy and cleaved PGAM5 promotes TFAM-mediated mitochondrial biogenesis and neurite re-growth **A** Expression of PGAM5 is up-regulated in injured neurons in response to TBI. Elevated PGAM5 enhances mitophagy in neurons. TBI causes loss of ΔΨ_m_ and the release of cleaved PGAM5 from mitochondria to cytosol. Cleaved PGAM5 enhances expression of TFAM and mitochondrial biogenesis followed by neurite re-growth and recovery of motor function deficit after TBI

Taken together, our results demonstrate that TBI induces transcriptional expression of PGAM5 and mitophagy. Mitophagy during acute phase, 24 h in our system, may facilitate removal of damaged mitochondria and trigger mitochondrial biogenesis during regeneration process. Damaged mitochondria also prompted PGAM5 being cleaved by PARL and subsequently increase mitochondrial biogenesis through increasing PGC1α and TFAM to promote neurite re-growth and functional recovery after TBI (Fig. 9).

## Discussion

Mitophagy are required to eliminate damaged mitochondria to maintain mitochondrial quality after TBI. PGAM5 was shown to stabilize PINK1 on mitochondria and activate mitophagy of damaged mitochondria [19, 24]. Consistent with this line of evidence, increased PGAM5 was shown to promote neuroprotection in mice models of Parkinson’s disease [47]. Our results demonstrate that TBI-induced up-regulation of PGAM5 is correlated with mitophagy in injured neurons (Fig. 5). However, overexpression of PGAM5 reduced neurite re-growth of injured cortical neurons. This result suggests that continuously increasing PGAM5 could be detrimental. In other words, requirement of mitophagy serves to maintain cellular mitochondrial quality at early-stage of TBI [14], but appropriate amount of mitochondria is required for neurite re-growth [12]. This is line with our finding that an obvious increase of PGAM5 was found after severe TBI, but not mild or moderated TBI (Fig. 1C, D).

While we observed a relatively low expression of PGAM5 in glia cells (Fig. 1F), consistent with a previous report of four times higher of PGAM5 in neuronal mitochondria compared to that in astrocytic mitochondria [48]. Nonetheless, we cannot exclude the possibility that PGAM5 in glia cells may indirectly regulate neuronal survival or neurite re-growth after TBI. It is reported that PGAM5 regulated neuroinflammation through activating microglia after TBI [49]. In microglia, PGAM5 enhanced microglial activation and secretion of inflammatory cytokine interleukin-1β (Il-1β) after TBI. TBI-induced secretion of Il-1β was inhibited in *Pgam5*^−/−^ mice. These results suggest that PGAM5 regulates neuroinflammation in microglia after TBI. A previous study demonstrated that administration of Il-1β promoted neurite growth in organotypic brain slices [50], suggesting that PGAM5-induced secretion of Il-1β in microglia may regulate neurite re-growth. Neuroinflammation also regulates PGAM5 expression reciprocally. TBI induces the expression of pro-inflammatory cytokine interferon-β (IFN-β), which induced microglial activation and neuroinflammation in brain [51]. IFN-β also enhanced PGAM5 expression to induce mitophagy in primary cortical neurons [47]. Whether and how the interplay between PGAM5 and neuroinflammation affects neurite re-growth remain to be elucidated.

Emerging evidence implicate that transplantation of healthy mitochondria restores mitochondrial function and promotes neurite re-growth after TBI [12, 52, 53]. However, mechanisms underlie mitochondrial biogenesis upon TBI are not clear. In this study, we showed that cleaved PGAM5 increased expression of TFAM and thus enhanced mitochondrial biogenesis (Fig. 7) and neurite re-growth of injured cortical neurons (Fig. 4B, C). The increased neurite re-growth is regulated by the activity of PARL, but not expression level of PARL. The catalytic activity of PARL is mainly regulated by phosphorylation of PARL at Ser-65, Thr-69, and Ser-70 [40]. While constitutively active PARL is beneficial for neurite re-growth (Fig. 4B, C), there is no specific activator of PARL to date. PARL is phosphorylated and activated by pyruvate dehydrogenase kinase 2 (PDK2), which is increased after TBI [39, 54]. Thus, a putative possibility is that PDK2 activators, such as NADH and acetyl-CoA [55], may increase PARL activity and thus PGAM5 cleavage and potentially neurite re-growth.

We took an approach to induce PGAM5 cleavage through challenging mitochondrial integrity using an uncoupler FCCP. Based on our findings that low-dose FCCP induces PGAM5 cleavage, promoted mitochondrial biogenesis, and restored motor function of CCI mice (Fig. 8 A-J), we proposed that induction of PGAM5 cleavage is a potential strategy to promote neurite re-growth and functional recovery after TBI. Given that full-length PGAM5 induces mitophagy, it is likely that full-length PGAM5 is needed to eliminate damage mitochondria at acute phase of TBI. At a later-stage of TBI, induction of PGAM5 cleavage enhances mitochondrial biogenesis and neurite re-growth. Thus, a timely regulation of PGAM5 expression and cleavage warrants a good outcome after severe TBI.

A recent study reported that PGAM5 was also up-regulated in kainate-induced epilepsy model [56]. Knockdown of PGAM5 suppressed neuronal damage of epileptic mice. It was also demonstrated that neuroinflammation and neuronal damage were reduced in *Pgam5*^*−/−*^ mice after TBI [49]. In contrast, PGAM5 deficiency induced Parkinson’s-like movement disorder in aged-mice and increased neurological deficits after ischemic injury [25, 57]. These studies demonstrate that the expression of PGAM5 has different effects in neurological diseases. However, current studies about neurological diseases only focused full-length PGAM5, while the role of cleaved PGAM5 in neurological diseases remains unknown. Given that mitochondrial transplantation is beneficial for neurological diseases such as Parkinson’s disease and ischemic stroke [58, 59], it is probably that induction of PGAM5 cleavage may also be beneficial for neurological diseases other than TBI.

## Conclusions

This study shows that severe TBI increases expression of PGAM5 through activating a novel enhancer-promoting interaction. Increased PGAM5 enhances mitophagy, whereas cleaved PGAM5 is released from mitochondria to cytosol to promote mitochondrial biogenesis and neurite re-growth of injured neurons. Induction of PGAM5 cleavage enhances mitochondrial biogenesis and facilitates recovery of motor function deficit and walking coordination of CCI mice (Fig. 9).

## Methods

### Controlled cortical impact

C57BL/6J male mice (8- to 10-weeks-old) were purchased from National Laboratory Animal Center (Taiwan). After anesthesia, a craniotomy was made to open a hole with a diameter of 3 mm in the left hemisphere. Then, the sensorimotor cortex (0.5–2.5 mm caudal to bregma and 0.5–2.5 mm lateral to the midline) was impacted with electric cortical contusion impactor (Custom Design & Fabrication, Inc., USA). The diameter of impact tip was 2 mm and impact dwell time was 250 ms. Mild, moderate and severe TBI were induced with following conditions: mild TBI (impact velocity of 3 m/s, impact depth of 1 mm), moderate TBI (impact velocity of 4 m/s, impact depth of 1.5 mm), and severe TBI (impact velocity of 5 m/s, impact depth of 2 mm). Sham mice underwent a craniotomy but were not impacted by CCI.

To measures tissue viability after TBI, the brain sections of CCI mice was stained with triphenyltetrazolium chloride (TTC) (Sigma-Aldrich). Briefly, mice were sacrificed and perfused intracardially with saline solution on 4 dpi. Brains were dissected and sliced into 1.5 mm coronal sections. Then, brain sections were incubated with 2% TTC solution for 15 min at 37℃.

For immunohistochemistry, the mice were perfused intracardially with saline solution and 4% paraformaldehyde (Alfa Aesar) solution on 4 dpi. The brains were dissected and immersed in 10%, 15%, and 20% sucrose sequentially to dehydrate the brains. Then the brains were embedded in tissue freezing medium (Leica) and sliced into sliced into 10-µm cryosections by cryostat microtome (Leica CM3050 S, Leica Biosystems, USA). The cryosections were air-dried at room temperature for 30 min and then stored in -80℃ for further immunohistochemistry.

To assess the expression of PGAM5 in the brains of CCI mice, mice were sacrificed and perfused intracardially with saline solution on 4 dpi. The left hemisphere was dissected and minced in SDS lysis buffer containing phosphatase inhibitors and protease inhibitors (240 mM tris–acetate, 1% SDS, 0.5% glycerol, 5 mM EDTA, 1 mM phenylmethanesulfonylfluoride, 1 mM sodium orthovanadate, 10 ng/ml aprotinin and leupeptin). Then the tissue was homogenized with a Dounce homogenizer, followed by centrifugation at 13,000 rpm for 15 min at 4 ℃. The supernatant was collected for immunoblotting.

### Primary glia and neuron culture

Pregnant Sprague–Dawley rats were purchased from BioLASCO Taiwan Co., Ltd. The brains of rat embryos (E18) were dissected and primary neurons were isolated and cultured in vitro as previous described [60]. For primary culture of glia cells, isolated cells were seeded on un-coated dishes on DIV0. On DIV1, suspended cells (neuron cells) were removed by change the medium to MEM (Thermo Fisher Scientific) containing 10% fetal bovine serum (SAFC Biosciences) and 1% penicillin/streptomycin (Thermo Fisher Scientific). The medium was changed every two days. On DIV8, primary neuron and glia cells were injured by scraping with a p20 pipette tip. Cells were harvested and lysed by SDS lysis buffer containing protease inhibitors and phosphatase inhibitors 24 h (DIV9) or 48 h (DIV10) after TBI.

### Western blotting

To assess protein level in brain or cells, lysates were prepared in SDS lysis buffer containing protease inhibitors and phosphatase inhibitors. The amount of proteins in lysates were normalized by BCA protein assay kit (Millipore). Proteins were resolved by SDS–polyacrylamide gel electrophoresis and then transferred to nitrocellulose membranes (PerkinElmer). Transferred blots were incubated with primary antibodies, anti-PGAM5 (1:500, Santa Cruz, SC-515880), anti-GAPDH (1:5000, Genetex, GTX100118), anti-TUJ1 (1:5000, BioLegend, #801202), anti-GFAP (1:5000, Genetex, GTX108711), anti-PGC1α (1:500, Genetex, GTX37356), anti-NRF1 (1:1000, Genetex, GTX103179), anti-TFAM (1:500, Genetex, GTX59889), or anti-TIM23 (1:500, Santa Cruz, SC-514463) overnight. Then the blots were incubated with secondary antibodies, IRDye 800CW goat anti-rabbit IgG secondary antibody (1:1000, LI-COR, # 926-32211) or goat anti-mouse IgG secondary antibody Alexa Fluor™ 700 (1:1000, Invitrogen, # A-21036), for 1 h. Membranes were imaged by ChemiDoc™ MP Imaging System (Bio-Rad) and the signal intensity of bands was quantified by Image Lab software (Bio-Rad, version 6.1.0) (Additional file 2).

### Semi-quantitative PCR (qPCR)

Total RNAs were extracted from control and injured cortical neurons on DIV9, followed by reverse transcription polymerase chain reaction. The transcript of *Pgam5* was analyzed using Power SYBR^®^ Green PCR Master Mix with StepOne Plus Real Time PCR System (Applied Biosystems). Relative *Pgam5* level in injured cortical neurons was normalized to the transcript of *Gapdh* and compared to control cortical neurons.

### Enhancer prediction

Putative enhancers were predicted as previously described [31]. Chromatin immunoprecipitation-sequencing datasets were downloaded from the ENCODE portal (https://www.encodeproject.org/) with the following identifiers: ENCFF033MMS, ENCFF247XHY, and ENCFF826KEG, and visualized on UCSC Genome Browser (https://genome.ucsc.edu/).

### Quantification of enhancer RNA

The transcript of enhancer RNA was assessed as previously described [31]. Total RNA was isolated from cortical neurons on DIV9, followed by reverse transcription polymerase chain reaction. Then, eRNAs were amplified by specific primers and analyzed by electrophoresis. The signal intensity of bands was quantified by Gel-Pro Analyzer 3.1 software. The intensity of eRNAs in injured cortical neurons was normalized to the transcript of *Gapdh* and compared to un-injured cortical neurons.

### Chromosome conformation capture analysis

Chromosome conformation capture (3C) assays were performed as previously described [31]. 3C template was collected from cortical neurons on DIV9. Then, the samples were digested with EcoRI (NEB), followed by ligation using T4 ligase (NEB). Ligation products were amplified by specific primers and analyzed by electrophoresis. The signal intensity of bands was quantified by Image Lab software (Bio-Rad, version 6.1.0). Amplifying primer sequences are listed in Table 1.

**Table 1:** Primer sequences used in this study

Primer sequences for eRNA		
Primer	Sense	Sequence
*Gapdh* (ctrl)	Sense	AAGGGCTCATGACCACAGTC
*Gapdh* (ctrl)	Antisense	TGTGAGGGAGATGCTCAGTG
e1-1	Sense	CAATTAGAGGCAGGCAGAGTAG
e1-1	Antisense	AAACATAAGGCTTACCCCAGAC
e1-2	Sense	CTGATTGCTGACTGGTGCTT
e1-2	Antisense	ACAGAAGGCTGGAGACACAA
e1-3	Sense	CCACAGAAGGTCAGAGGTCA
e1-3	Antisense	CCAGGCTAGGTTGGGATAAC
e1-4	Sense	GCTGCGTAAGAGTGACAGGA
e1-4	Antisense	AAGTGCTCAGGTAGGGAAGC
e1-5	Sense	GGATGGGTAGGGGGTACATA
e1-5	Antisense	GGGGAATGGATGCTAGTAGG
e2-1	Sense	GTCACGAGTGCGACTACACT
e2-1	Antisense	ACTCACCTTGACCTGGAAGG
e2-2	Sense	ACCTACCTCGGTCTCTGAGT
e2-2	Antisense	TCATAACCACCAGACGCCTt
e2-3	Sense	TGTATGTTACGGGGTGGAGC
e2-3	Antisense	AGACCTGGGTTTGGTTCACA
e2-4	Sense	TTCATCAAGGAGGGACTGGG
e2-4	Antisense	CAAAGGAGGAACAGGTTGTGG
e2-5	Sense	TTGGAGATTCCTTCTCGCCA
e2-5	Antisense	AGGACAGCAGTCTACAGTGG
e2-6	Sense	AGAGCCTAGCAGCGAAGATT
e2-6	Antisense	TAGGGTTACGAAGCCTGAGC
e3-1	Sense	TTACACCAGGGAGCAGGAG
e3-1	Antisense	CAGACCCGTGGAAATCTTG
e3-2	Sense	ACCCAACCAGTGGACAGAA
e3-2	Antisense	CTCCTCCCAGTTCGCTCTA
e3-3	Sense	TTTAGATGTGGCGTCTCCAG
e3-3	Antisense	CCTTCAGCCTTCATTTACCAA
e3-4	Sense	CCACCTGTCTCACTGCTGTT
e3-4	Antisense	CCATGTAGCCTCCTGCTGTA
e3-5	Sense	ACACGCATACGCACACTCTT
e3-5	Antisense	AGGTTGCCATTTTGCTGAAGT
e3-6	Sense	GTCAAGCTACATCCAGTAAAGCT
e3-6	Antisense	TGGCTTCATGGTTCCTGTC
e3-7	Sense	CCAGGAGCCTGAAAGCTATG
e3-7	Antisense	ACTCATCAGCCAGTGTCACG
e4-1	Sense	GACTGTCTTTCCCACAGCAA
e4-1	Antisense	GCACACTTAGCCAAGCACAT
e4-2	Sense	GCTAAGTGGCTCAGAGAATGG
e4-2	Antisense	CCTAAATGGGTTTCCTGTGAA
e4-3	Sense	TTCTGCTTGGGTAAGGGAAG
e4-3	Antisense	TATGTGAAAGCGGATTGGAG
e4-4	Sense	AAGTTGCCCATAGGACAGGA
e4-4	Antisense	TTCCCAGTTCTGACCCAGAT
e4-5	Sense	TCATAAAGCCAGGCGATACAC
e4-5	Antisense	AGGGTCTCTTGGGATTCTCC
e4-6	Sense	TCGGAGTTCACACCAAACAC
e4-6	Antisense	GAGATGACCCATCCCTTCAG
e4-7	Sense	CATACACTCGGGCACTCAGT
e4-7	Antisense	GGTCTGGGTATTTGAAATCAGG
e4-8	Sense	CCACTGCTGTCACATTCCAT
e4-8	Antisense	CGTTCTGAATGGTTGCCAAG
e4-9	Sense	GAGATGCTCTCCCTCCAGAA
e4-9	Antisense	TCAGAGTCTGTGCCCACTGT
e5-1	Sense	CCAGTGTTTCAACCCTGATGT
e5-1	Antisense	AATTTCACGGATGGAGGAAGC
e5-2	Sense	CGGGCACCCAGTTAATTCAT
e5-2	Antisense	TCACCCCATCACAAGAATGC
e5-3	Sense	AGGATCAGGTTGTTGGTCTG
e5-3	Antisense	GGCAAGGACGAATGTAAGCA
e5-4	Sense	CCCTGCTAACCATTAGTGCC
e5-4	Antisense	TGAAGTTTGAGCCCACTTGAC
e5-5	Sense	CTGGCTCCTCTAACCTTTCGT
e5-5	Antisense	GAACACTCCCTAGAGTTTCCCTA
e5-6	Sense	CTGTCTTCAATCTCAAAGGGG
e5-6	Antisense	CTGTCTGTCATCCACGCAAT
e6-1	Sense	AACCGCATATACCCCGAAGA
e6-1	Antisense	TGTGATACTTGGACGGCAGA
e6-2	Sense	CAGACTAGGTCAGGCACCAA
e6-2	Antisense	TGACATCCATCCCATGCGTA
e6-3	Sense	AATGCTAACCAAGTCGCTGC
e6-3	Antisense	TATCCGACACCTTCACCCAC
e6-4	Sense	CCCCGCCCCCATTATCTTAT
e6-4	Antisense	TGAGTCCCACAACATCGGAA
e6-5	Sense	ATAGAAGTGTGAGTGCCCCC
e6-5	Antisense	CATCACCACGGATGCCAAAT
e6-6	Sense	AGCCTTAAATAACGCCCCCT
e6-6	Antisense	AGCATTGTTCTTTGCCCCAG
e6-1-a	Sense	TCCACAAGAAGCTCCCAGA
e6-1-a	Antisense	AAATACCTCCTGCTCGGCTT
e6-1-b	Sense	GCAATGGTGTCCAAGAGTCA
e6-1-b	Antisense	TGTGTTTGGTCAGGTCTCC
e6-1-c	Sense	GCTCTGGCTCACTGAAGTCT
e6-1-c	Antisense	GAACAGTCTTGGGTGTTGCG
e6-1-d	Sense	AAGACTGGAGACCAGGCAAT
e6-1-d	Antisense	GGGAATGGGAACGACATCT
e6-1-e	Sense	CGTCTGGCTCAGAGAGGATT
e6-1-e	Antisense	TACCATGAACTCCCCACCTG
Primer sequences for 3C assays		
Loading control	Sense	TTCTTGGGGTGAAGCAACACAT
Loading control	Antisense	ACCAGAGCAGGACCTGTTAAATG
e6-1-a	Sense	AGAAGCATTGACAAGCTCCGC
*Pgam5* promoter	Antisense	GATCATGGACAGGGTAGGCAG
Primer sequences for enhancer-reporter constructs		
e6-1-a (AseI-NdeI)	Sense	TTATTAATTGGTGTGTTCGCCTTGGAAT
e6-1-a (AseI-NdeI)	Antisense	TTCATATGCTGTCCACCCTGGCATTTCT
*Pgam5* promoter (NdeI-AgeI)	Sense	ATCATATGCCCTTGCCACATCCCTTTTC
*Pgam5* promoter (NdeI-AgeI)	Antisense	TTACCGGTTTTCCCCGAAACAGCAGGAA
*Pgam5* promoter (AseI-AgeI)	Sense	ATATTAATCCCTTGCCACATCCCTTTTC
*Pgam5* promoter (AseI-AgeI)	Antisense	TTACCGGTTTTCCCCGAAACAGCAGGAA
Primer sequences for qPCR		
*Pgam5*	Sense	CCACCTGTGTCTCACTGGAAGC
*Pgam5*	Antisense	ACGGATGACATTGGCGTGACAT
*Gapdh*	Sense	ATGACTCTACCCACGGCAAGTT
*Gapdh*	Antisense	TCCCATTCTCAGCCTTGACTGT

### Enhancer reporter assay

Neuro2a cells (2 × 10^6^ cells) were transiently transfected with pPgam5-GFP or e6-1-a-pPgam5-GFP vectors. The pmCherry-C2 vector was co-transfected to neuro2a cells as a reference of transfection efficiency (the ratio of reporter vector to mCherry is 5:1). GFP fluorescence represents the relative transcriptional activity. Bright view images showed relative cell numbers in the fields. Images were taken using Carl Zeiss Observer Z1 microscope 24 h after transfection.

### DNA constructs

pcDNA3 PARL-FLAG-CT wild type (Addgene plasmid # 13639), pcDNA3 PARL-FLAG-CT S65A + T69A + S70A (Addgene plasmid # 13616), pcDNA3 PARL-FLAG-CT S65D + T69D + S70D (Addgene plasmid # 13617) were gifts from Luca Pellegrini [38, 61]. The pmCherry-C2 construct was a gift from Dr. Lily Hui-Ching Wang at National Tsing Hua University, Taiwan. Knockdown constructs, shLacZ, shPgam5, and shParl constructs, were purchased from the National RNAi Core Facility of Academia Sinica (Taipei, Taiwan). Full-length PGAM5 and PGAM5(Δ2-24) were both subcloned into pEGFP-C1 construct via NheI-AgeI sites. For enhancer-reporter constructs, e6-1-a and *Pgam5* promoter were amplified from the RNA and genomic DNA isolated from rat cortical neurons, respectively. For pPgam5-GFP construct, *Pgam5* promoter fragment was subcloned into pEGFP-C1 construct via AseI-AgeI sites. For e6-1-a-pPgam5-GFP construct, e6-1-a and *Pgam5* promoter fragments were subcloned into pEGFP-C1 construct via AseI-NdeI and NdeI-AgeI sites, respectively. Specific primers used to amplify e6-1-a and *Pgam5* promoter are listed in Table 1. All constructs were transfected in cells using Lipofectamine 2000 (Invitrogen) in accordance with its protocol.

### Injury assays of cortical neurons

Cortical neurons (7.5 × 10^5^ cells/ml) were cultured in 6-well plate on DIV0. Cortical neurons were transiently transfected with PGAM5, PGAM5(Δ2-24), hPARL, hPARL(AAA), hPARL(DDD), shlacZ, shPgam5, or shParl constructs on DIV7. To visualize neurites, cells were co-transfected with EGFP-C2 construct. On DIV8, cortical neurons were scratch-injured with a p20 pipette tip. Cortical neurons were imaged using Carl Zeiss Observer Z1 microscope on DIV9. The length of re-growing neurites was measured using ImageJ software (plugins NeuronJ).

### Measurement of mitophagy

Mitophagy was assessed by the co-localization of mitochondria and lysosomes in hippocampal neurons [62]. Hippocampal neurons (4 × 10^4^ cells/ml) were cultured in 2-well chamber slices (Thermo Fisher Scientific). On DIV7, hippocampal neurons were transiently transfected with PGAM5, PGAM5(Δ2-24), hPARL(AAA) or hPARL(DDD) constructs. To visualize mitochondria, hippocampal neurons were transfected with MitoGFP. On DIV9, hippocampal neurons were incubated in culture medium containing 75 nM LysoTracker™ Red DND-99 (Invitrogen) for 45 min at 37℃. Then, hippocampal neurons were imaged using Carl Zeiss LSM800 confocal microscope. The co-localization of mitochondria and lysosomes were assessed using a custom-written MATLAB code in conjunction with the Image Processing Toolbox of MATLAB (version R2021b). MitoGFP^+^ and LysoTracker^+^ area were obtained according to specific criteria: MitoGFP intensity > 80 and area > 0.1 μm^2^; LysoTracker intensity > 60 and area > 0.05 μm^2^. Then, co-localization% of mitochondria and lysosomes was quantified as MitoGFP^+^ LysoTracker^+^ area divided by MitoGFP^+^ area.

### Measurement of ΔΨ_m_

To investigate the change of ΔΨ_m_ after TBI, hippocampal neurons (4 × 10^4^ cells/ml) were cultured in 2-well chamber slices. Hippocampal neurons were scratch-injured with a p2 pipette tip on DIV8. On DIV9 and DIV10, hippocampal neurons were incubated in culture medium containing 250 nM tetramethylrhodamine (Invitrogen) for 30 min at 37℃, followed by washed twice with PBS. Hippocampal neurons were imaged using Carl Zeiss Observer Z1 microscope. The intensity of TMRM were quantified by a custom-written MATLAB code in conjunction with the Image Processing Toolbox of MATLAB (version R2021b).

### Immunostaining

Immunohistochemistry was performed as previously described [30]. Briefly, cryosections were incubated in antigen retrieval solution (Nacalai Tesque) at 70℃ for 20 min to unmask antigenic sites. After incubated in blocking buffer containing 1% BSA (Sigma-Aldrich) for 2 h, cryosections were incubated in 1% BSA containing anti-NeuN antibody (1:500, Genetex, GTX132974) overnight at 4℃. Finally, cryosections were incubated in goat anti-rabbit secondary antibody (1:500, Invitrogen, A21428) for 1 h and imaged using Carl Zeiss LSM800 confocal microscope.

To investigate the sub-cellular location of PGAM5, PGAM5 and mitochondria were visualized using immunostaining. Hippocampal neurons (4 × 10^4^ cells/ml) were cultured on cover glass (Marienfeld) and injured on DIV8. Then, hippocampal neurons were fixed with 4% paraformaldehyde and permeabilized with 0.1% Triton X-100 (Sigma-Aldrich) on DIV9 or DIV10. After incubated in 1% BSA for 1 h, cells were incubated with anti-PGAM5 (1:100, Santa Cruz, SC-515880) and anti-TOM20 (1:100, Santa Cruz, SC-17764) antibodies, overnight at 4 ℃. Then, cells were incubated with secondary antibodies, goat anti-mouse secondary antibody (1:1000, Invitrogen, A11001) and goat anti-rabbit secondary antibody (1:1000, Invitrogen, A21428), for 1 h at room temperature. Finally, the cells were mounted in ProLong™ Gold Antifade Mountant (Invitrogen) and imaged using Carl Zeiss LSM800 confocal microscope. The co-localization of PGAM5 and TOM20 were assessed using a custom-written MATLAB code in conjunction with the Image Processing Toolbox of MATLAB. PGAM5^+^ and TOM20^+^ area were obtained according to specific criteria: PGAM5 intensity > 40; TOM20 intensity > 60. Then, the percentage of mitochondrial PGAM5 was quantified as PGAM5^+^TOM20^+^ area divided by PGAM5^+^ area.

### Measurement of mitochondrial mass

To evaluate mitochondrial mass after TBI, hippocampal neurons (4 × 10^4^ cells/ml) were cultured in 2-well chamber slices. Hippocampal neurons were injured on DIV8. On DIV9 and DIV10, hippocampal neurons were incubated in culture medium containing 500 nM MitoTracker Red (Invitrogen) for 30 min at 37℃. After washed twice with culture medium, hippocampal neurons were imaged under Carl Zeiss Observer Z1 microscope. Total intensity of MitoTracker Red in individual images were obtained using a custom-written MATLAB code in conjunction with the Image Processing Toolbox of MATLAB.

Neuro2a cells (1 × 10^5^ cells/ml) were incubated in 90% MEM (Thermo Fisher Scientific), supplemented with 2 mM L-glutamine (Thermo Fisher Scientific), 1.5 g/L sodium bicarbonate (Thermo Fisher Scientific), 0.1 mM non-essential amino acids (Thermo Fisher Scientific), 1.0 mM sodium pyruvate (Thermo Fisher Scientific), and 10% fetal bovine serum. To assess mitochondrial mass in neuro2a cells, neuro2a cells were incubated in culture medium containing 0.1 μM MitoBright LT Deep Red (Dojindo) for 15 min at 37℃. After be washed twice with culture medium, neuro2a cells were imaged using Carl Zeiss LSM800 confocal microscope. Total intensity of MitoBright LT Deep Red in individual cells were obtained using MATLAB.

### FCCP administration

Neuro2a cells (2 × 10^5^ cells/ml) were treated with 0.1 μM FCCP (Sigma-Aldrich), 1.0 μM FCCP or 0.1% DMSO (Ctrl). Cells were harvested by SDS lysis buffer containing protease inhibitors and phosphatase inhibitors 24 h after treatment. The proteins were further analyzed using immunoblotting.

For CCI mice, FCCP was administrated intranasally 6 h after CCI as previously described [63]. Intranasal administration allows drugs to bypass blood–brain barrier and increase brain bioavailability [63, 64]. FCCP or DMSO (vehicle) was diluted in 24 μl saline. Saline containing FCCP or DMSO was intranasally administrated in 2 rounds. In the first round, mouse was intranasally administrated 6 μl saline to the left nostril. Then the mouse was held 15 s to confirm the saline was fully administrated into nose, followed by an administration of 6 μl saline to the right nostril. After a 2-min rest, the second round was performed. The total of 24 μl saline was administrated 6 h after CCI.

### Rotarod test

To evaluate motor coordination of CCI mice, rotarod test was performed as previously described [45]. To perform the pre-training trial, mice were placed on the rod (Ugo-Basile, Italy, #47650) rotating at 4 revolutions per minute (rpm) for 60 s on 1 dpi. Then mice were placed on a rotating rod accelerating from 4 to 40 rpm over 3 min and the latency to fall was recorded. Mice were tested 3 times a day for 1–4 dpi and there was a 10-min rest between each trial. The average latency to fall for the 3 trials was recorded.

### Grid test

To evaluate spontaneous motor deficits of CCI mice, grid test was performed as previously described [46]. Mice were placed on an elevated steel grid with dimensions of 35 × 20 cm and with grid size of 1.1 × 1.1 cm. Mice were allowed to walk around for 5 min. A foot fault is defined as a paw missed a wire edge or slipped off. Foot faults of each limb in 5 min were recorded. Grid test was performed on -1 dpi to assess basal motor function before CCI. After CCI, grid tests were performed on 1, 3, 6 dpi to evaluate motor deficit.

### Statistical analysis

All results are expressed as mean ± SEM or violin plot from at least three independent experiments. Data were analyzed by paired or unpaired two-tailed Student’s t-tests or ANOVA with Dunnett’s multiple comparisons or Tukey’s multiple comparisons using Prism software. Statistical significance is defined as *p* < 0.05.

## Supplementary Information

**Additional file 1: Figure S1**. Relative *Pgam5* level in injured cortical neurons. Total RNAs were isolated from control and injured cortical neurons on DIV9 and *Pgam5* transcript was analyzed with qPCR. Relative *Pgam5* transcript in injured cortical neurons was normalized to that in control neurons. Data are presented as mean ± SEM.

**Additional file 2:**Immunoblots of protein expressions in mouse brain tissue, cortical neurons, and neuro2a cells.

## Abbreviations

TBI: Traumatic brain injury
PGAM5: Phosphoglycerate mutase 5
PARL: Presenilins-associated rhomboid-like protein
TFAM: Mitochondrial transcription factor A
TTC: Triphenyltetrazolium chloride
FCCP: Carbonyl cyanide 4-(trifluoromethoxy) phenylhydrazone
ΔΨ_m_: Mitochondrial membrane potential
DRP1: Dynamin-related protein 1
PGC1α: Peroxisome proliferator-activated receptor gamma coactivator-1 alpha
PINK1: Phosphatase and tensin homolog-induced putative kinase protein 1
3C: Chromosome conformation capture
CCI: Controlled cortical impact
DIV: Day in vitro
H3K27ac: Histone H3 lysine 27 acetylation
H3K4me3: Histone H3 lysine 4 tri-methylation
H3K4me1: Histone H3 lysine 4 monomethylation
eRNA: Enhancer RNA
hPARL(AAA): Human PARL(S65A/T69A/S70A)
hPARL(DDD): Human PARL(S65D/T69D/S70D)
NRF1: Nuclear respiratory factor 1
Il-1β: Interleukin-1β
IFN-β: Interferon-β
PDK2: Pyruvate dehydrogenase kinase 2
