# Supplementary figures and images for: Timely expression of PGAM5 and its cleavage control mitochondrial homeostasis during neurite re-growth after traumatic brain injury
Source: Cell Biosci. 2023 May 23;13:96. doi: 10.1186/s13578-023-01052-0 (PMC10207772; doi:10.1186/s13578-023-01052-0)

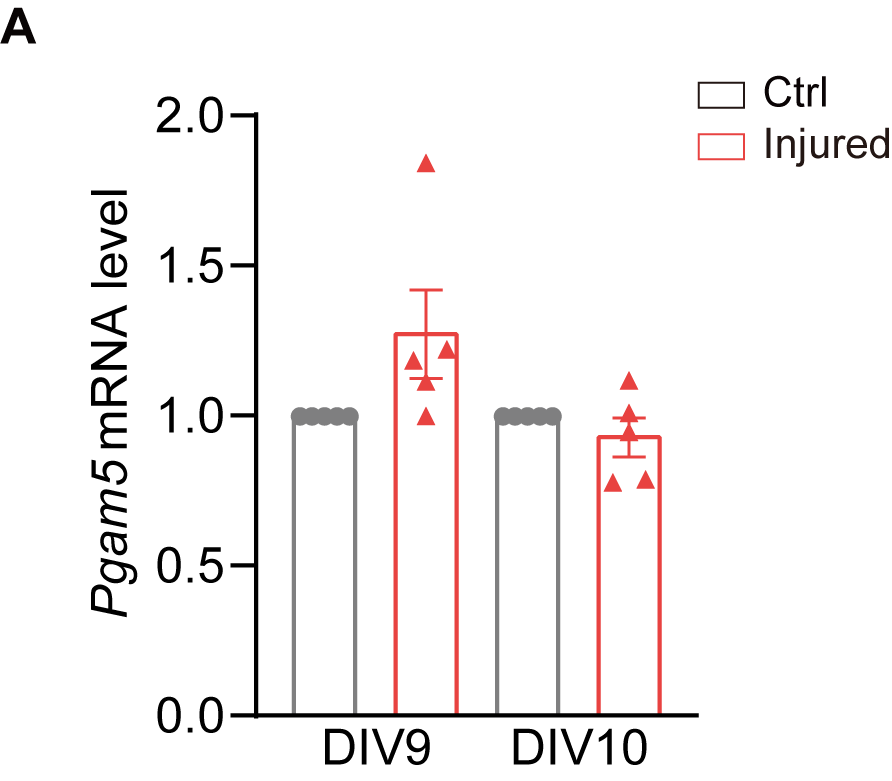

Supplement: Supplementary file 1 — Additional file 1: Figure S1. Relative Pgam5 level in injured cortical neurons. Total RNAs were isolated from control and injured cortical neurons on DIV9 and Pgam5 transcript was analyzed with qPCR. Relative Pgam5 transcript in injured cortical neurons was normalized to that in control neurons. Data are presented as mean ± SEM. [file 13578_2023_1052_MOESM1_ESM.tif]
