# Supplementary material for: Timely expression of PGAM5 and its cleavage control mitochondrial homeostasis during neurite re-growth after traumatic brain injury
Source: Cell Biosci. 2023 May 23;13:96. doi: 10.1186/s13578-023-01052-0 (PMC10207772; doi:10.1186/s13578-023-01052-0)
Supplement: Supplementary file 2 — Additional file 2:Immunoblots of protein expressions in mouse brain tissue, cortical neurons, and neuro2a cells. [file 13578_2023_1052_MOESM2_ESM.pdf]

Additional file

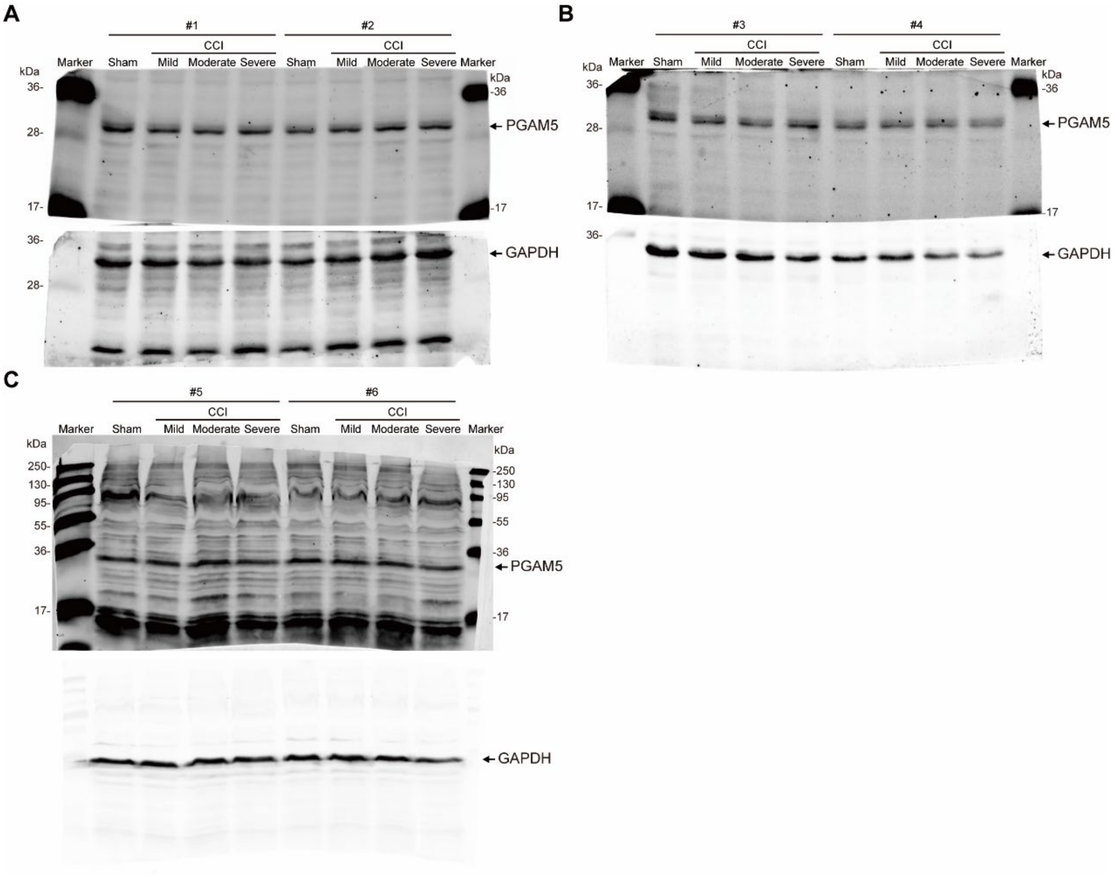

Immunoblots of PGAM5 in mice brain tissue on 4 dpi

(A-C) Individual immunoblots of PGAM5 and GAPDH in brain tissue lysate collected from left hemisphere on 4 dpi. Arrows indicate the specific bands of PGAM5 and GAPDH.

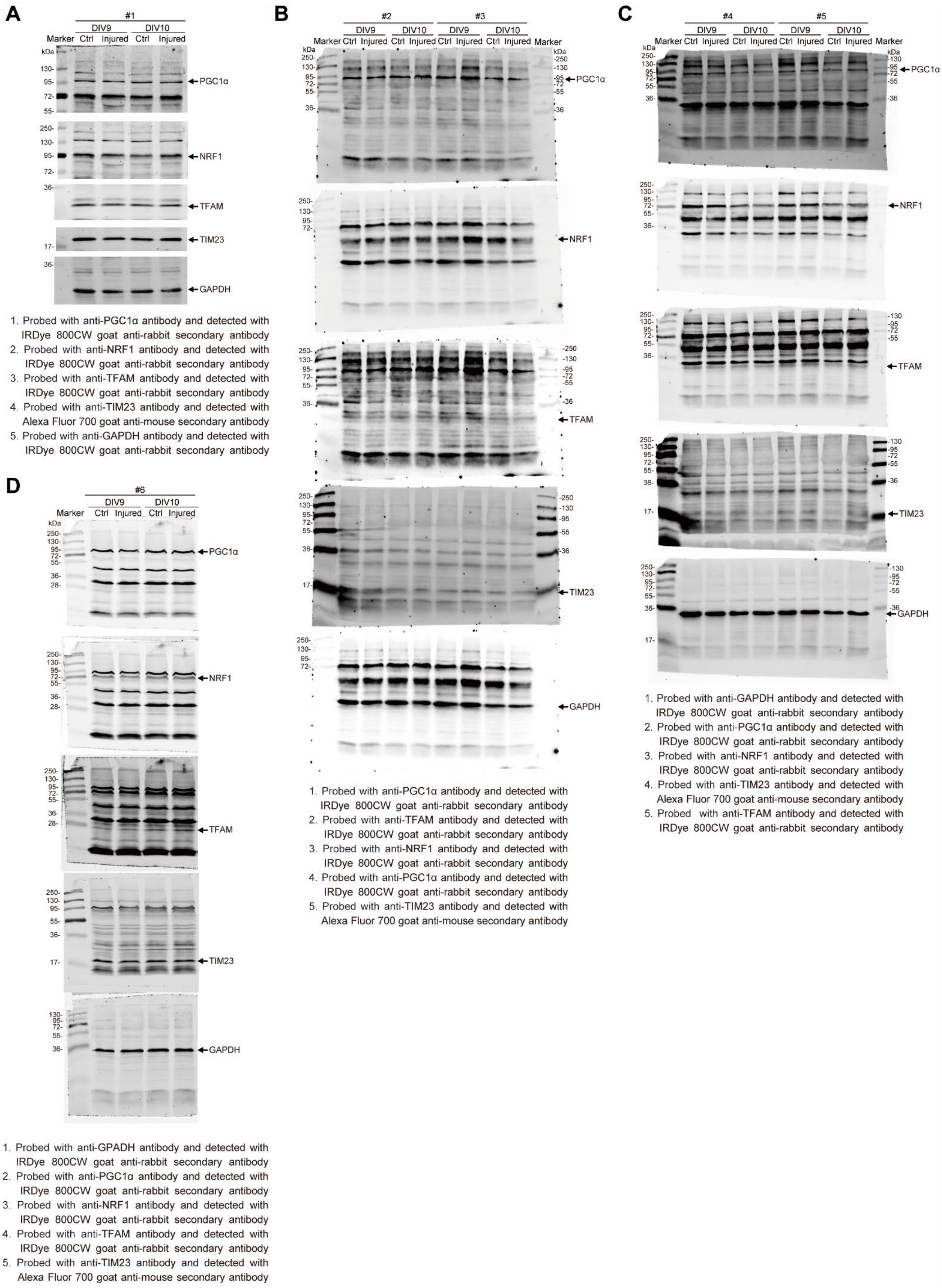

Immunoblots of PGC1α, NRF1, TFAM and TIM23 in cortical neurons

(A-D) Individual immunoblots of PGC1α, NRF1, TFAM, TIM23 and GAPDH in control and injured cortical

neurons. Arrows indicate the specific bands of PGC1α, NRF1, TFAM, TIM23 and GAPDH. The immunoblots were probed with antibodies in the order listed under the immunoblots.

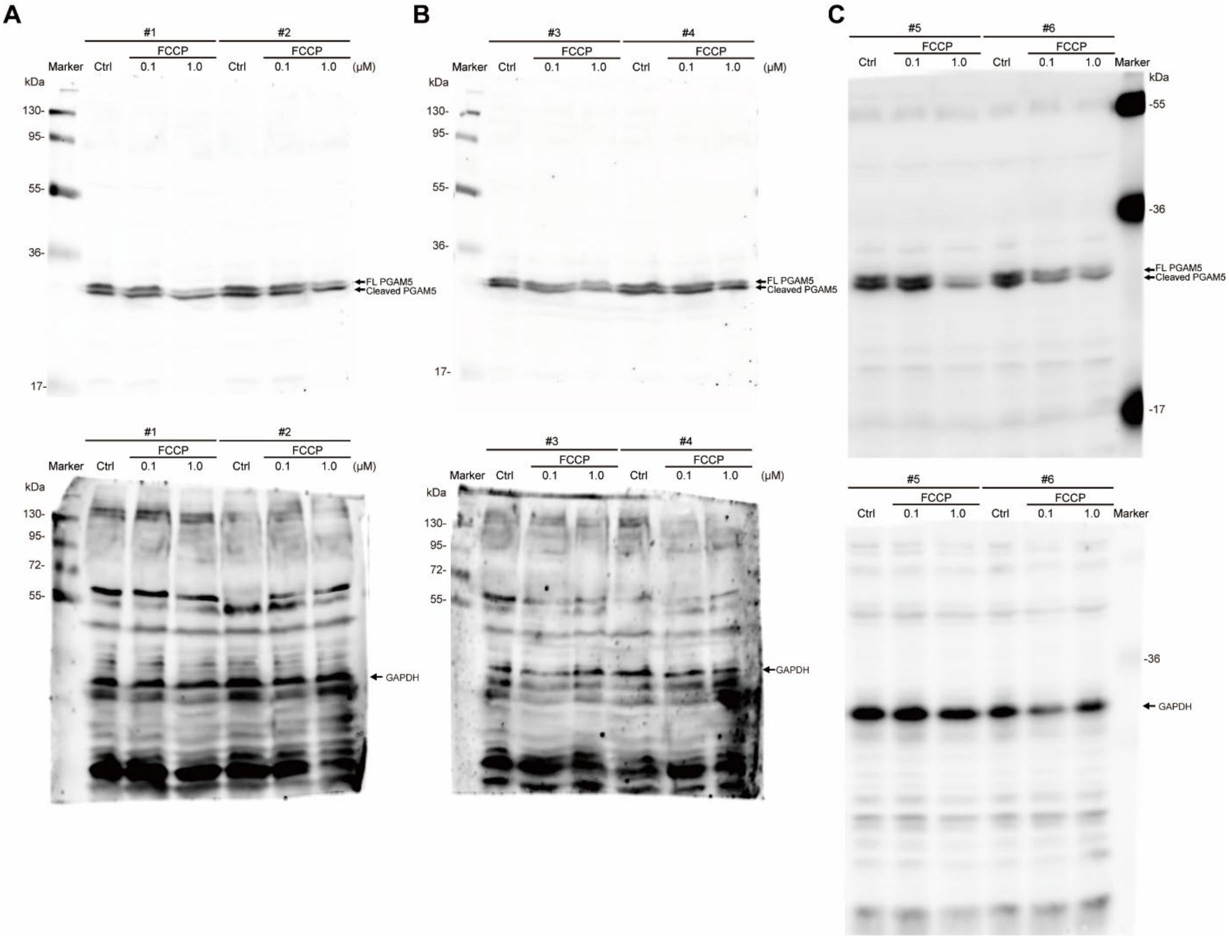

Immunoblots of PGAM5 in neuro2a cells

(A-C) Individual immunoblots of PGAM5 and GAPDH in neuro2a cells treated with DMSO, 0.1 μM FCCP and 1.0 μM FCCP. Arrows indicate the specific bands of full-length PGAM5, cleaved PGAM5 and GAPDH. FL: full-length.
